# Supplementary material for: Programming mechanics in knitted materials, stitch by stitch
Source: Nat Commun. 2024 Mar 23;15:2622. doi: 10.1038/s41467-024-46498-z (PMC10960873; doi:10.1038/s41467-024-46498-z)
Supplement: Supplementary file 1 — Supplementary Information [file 41467_2024_46498_MOESM1_ESM.pdf]

Supplementary Information for "Programming Mechanics in Knitted Materials, Stitch by Stitch"

Krishma Singal[1], Michael S. Dimitriyev[2,3], Sarah E. Gonzalez[1], A. Patrick Cachine[1], Sam Quinn[1], and Elisabetta A. Matsumoto[1,4*]

[1]*School of Physics, Georgia Institute of Technology, Atlanta, Georgia 30332, USA*

[2]*Department of Polymer Science and Engineering, University of Massachusetts, Amherst, Massachusetts 01003, USA*

[3]*Department of Materials Science and Engineering, Texas A&M University, College Station, Texas 77843, USA and*

[4]*International Institute for Sustainability with Knotted Chiral Meta Matter (WPI-SKCM), Hiroshima University, Higashihiroshima 739-8526, Japan*

(Dated: February 26, 2024)

SUPPLEMENTARY TEXT

SUPPLEMENTARY NOTE 1. Uniaxial stretching experiment

From the uniaxial stretching experiments, we extracted the average fabric response by expressing the applied force F_x (or F_y) in terms of stress components $\sigma_{xx} = F_x/W_y$ (or $\sigma_{yy} = F_y/W_x$), where W_x and W_y are fabric widths measured at the clamped edges.

To obtain the average strain response of the fabric, we focused on the displacement of four points placed on the axes of symmetry of the fabric. Two points were marked with pins located on the transverse axis and the other two points were marked by painted dots on the clamps along the direction of stretching. In the case where the fabric is stretched along the y-direction, two red points painted on the clamps are aligned such that the line connecting them lies along the y-axis and has length L_y. The other two pins were positioned close to the waist of the fabric such that the line connecting them lies along the x-axis and has length L_x. For the lace weight and the glove prototype samples, there are only two pins along the transverse axis. The clamp displacement is tracked via the UTM. The principal components of the strain tensor are then $\varepsilon_{xx} = (L_x - L_{x,0})/L_{x,0}$ and $\varepsilon_{yy} = (L_y - L_{y,0})/L_{y,0}$, where $L_{x,0}$ and $L_{y,0}$ are the respective pin separations of the un-stretched fabric (see Supplementary Fig. 1).

Figure Supplementary Fig. 2 shows the results of the stress-strain analysis for the cotton yarn.

* sabetta@gatech.edu

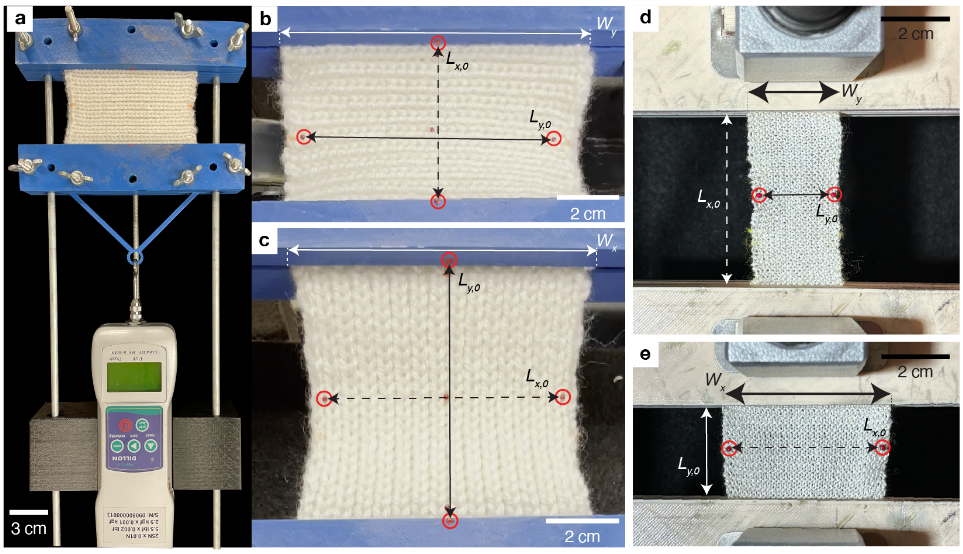

Supplementary Fig. 1. (**a**) Top view of the experimental setup. During experiments we control displacement while measuring force. We perform uniaxial stress-strain experiments with forces applied along the rows (**b**) and columns (**c**). A rib fabric being stretched along its (**b**) x- and (**c**) y-directions. The four points we tracked to characterize the bulk response of the fabric are circled in red. The initial pin separation along the x-axis is $L_{x,0}$ and along the y-axis is $L_{y,0}$. The values W_x and W_y are the widths of the fabric held down at the clamps. The lace weight and the therapeutic glove prototype samples underwent uniaxial stretching experiments on an Instron UTM. A bamboo garter sample stretched along its (**c**) x- and (**d**) y-axis. These tests only require two red pins to be tracked while the displacement between the clamps is tracked by the UTM software.

SUPPLEMENTARY NOTE 2. Measuring the bending modulus

The yarn of linear mass density λ and length L is adhered to the edge of a flat surface at $x = 0$ and $y = 0$ and the free end is allowed to drape under gravity. Equilibrium distributions of the internal moment $\mathbf{M}(s)$ and force $\mathbf{T}(s)$ are governed by the Kirchhoff rod equations [1],

$$\partial_s \mathbf{T}(s) - \lambda g \hat{\mathbf{y}} = 0\,, \qquad \text{(Supplementary Equation 1a)}$$

$$\partial_s \mathbf{M}(s) + \hat{\mathbf{t}}(s) \times \mathbf{T}(s) = 0\,, \qquad \text{(Supplementary Equation 1b)}$$

where $s \in [0, L]$ is the arclength coordinate with $s = 0$ at the fixed end and $s = L$ at the free end. Integrating Eqs. Supplementary Equation 1a, Supplementary Equation 1b and using the free end boundary conditions $\mathbf{T}(L) = \mathbf{M}(L) = 0$, we have

$$\mathbf{T}(s) = \lambda g (s - L) \hat{\mathbf{y}}\,, \qquad \text{(Supplementary Equation 2a)}$$

$$\mathbf{M}(s) = \lambda g \hat{\mathbf{y}} \cdot \int_s^L \mathrm{d}s'\, \hat{\mathbf{t}}(s')(s' - L)\,. \qquad \text{(Supplementary Equation 2b)}$$

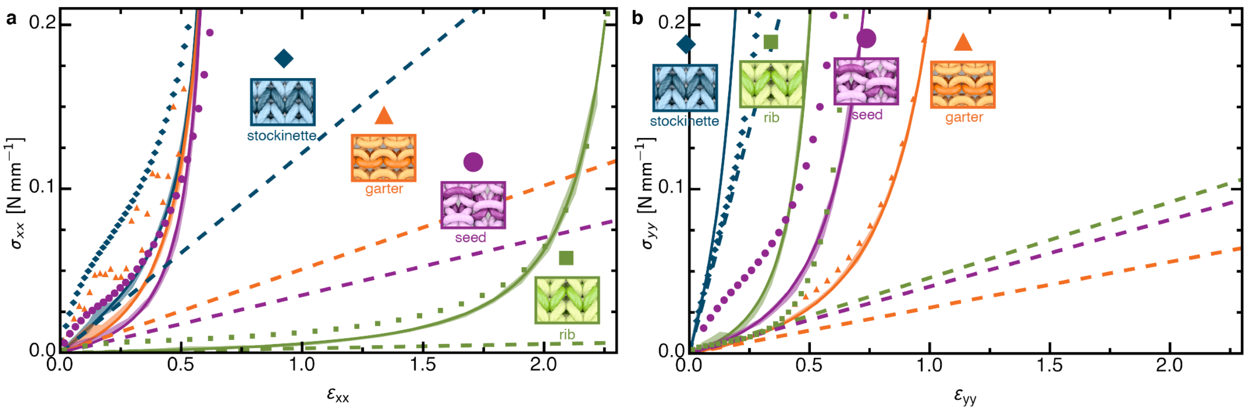

Supplementary Fig. 2. The stress-versus-strain relations for the four fabrics made from the cotton yarn in the (**a**) x- and (**b**) y-directions. All of the data for each type of fabric is displayed by a different color: stockinette in blue, garter in orange, rib in green, and seed in purple. The experimental data is shown in the translucent regions where the width of the region is one standard deviation of the four experiment runs. The simulation data is shown with solid symbols. The solid curves are fits to the constitutive relations. Dashed lines depict the linear response at zero stress. Experiments applying force in the x-direction show the extreme extensibility of the rib pattern compared with the other three. Garter and seed dominate in the y-direction.

Evaluating Supplementary Equation 2b at the clamping point $s = 0$ and integrating by parts, we find

$$\mathbf{M}(0) = -\lambda L g x^* \hat{\mathbf{y}}\,, \qquad \text{(Supplementary Equation 3)}$$

where $x^* = L^{-1}\int_0^L \mathrm{d}s\, x(s)$ is the x-component of the center of mass of the hanging yarn, recovering the basic result that the total gravitational torque applied to the yarn at the fixed boundary is simply the total gravitational force of the yarn, $-\lambda L g \hat{\mathbf{y}}$, times its lever arm x^*. Finally, assuming the linear constitutive relationship $M(s) = B\kappa(s)$, we solve for the bending modulus B of the yarn, viz.

$$B = \frac{\lambda L g x^*}{\kappa(0)}\,, \qquad \text{(Supplementary Equation 4)}$$

where $\kappa(0)$ is the curvature discontinuity of the yarn at the clamping point. We perform experiments on yarn samples of varying length (see Supplementary Fig. 4).

In order to extract the curvature discontinuity at the suspension point, we must determine the shape of the yarn in space. After an image is taken and cropped to the suspended yarn, the exposure is adjusted to maximize contrast between the yarn and the background. We apply a blur and binarize filter to the yarn images and fit the white pixels in the domain $y > -y_{\max}/5$ to the 4th-order polynomial curve $y(x) = (a/2)x^2 + (b/4)x^4$. This is reminiscent of how Cornelissen and Akkerman [2] used a polynomial fit to study yarn deflection during cantilever experiments. Images are blurred based on how many pixels out we can see stray fibers. For yarns containing certain fibers, stray filaments will inhomogeneously extend many yarn-radii from the spun center of the yarn. For these exceptionally fuzzy yarn types (alpaca mohair and blue mohair), the blur required to erase stray fibers is so great that a reliable fit to the core of the yarn is not feasible. In these cases, a simple blur filter is not

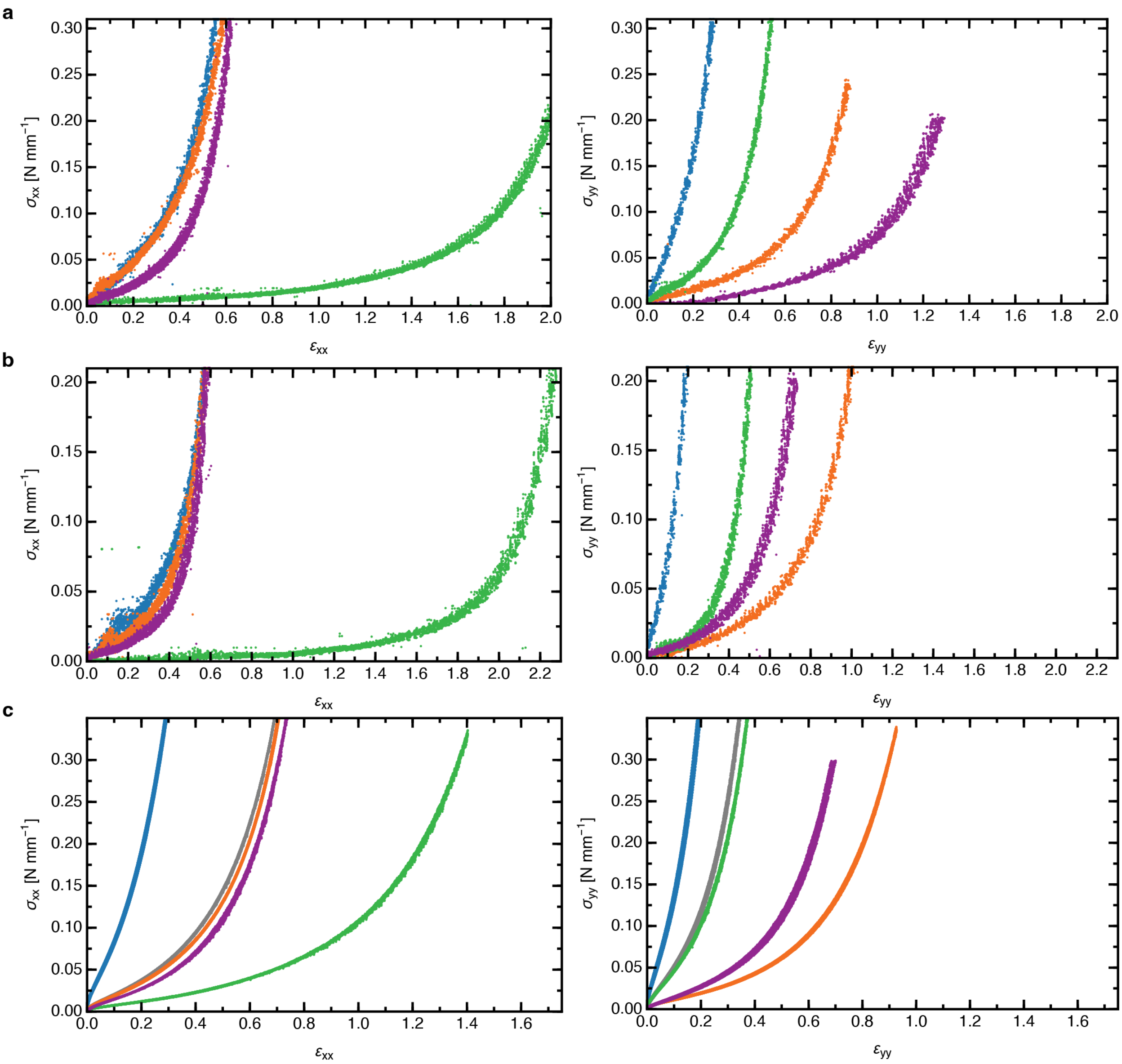

Supplementary Fig. 3. The raw stress-versus-strain experimental data for the (**a**) acrylic, (**b**) cotton, and (**c**), therapeutic glove samples. All of the data for each type of fabric is displayed by a different color: stockinette in blue, garter in orange, rib in green, and seed in purple. For the therapeutic glove, there is an additional experimental data set shown in gray for a stockinette sample made with 2.75mm knitting needles.

sufficient in isolating the core and, after adjusting the exposure, the outer halo of the yarn was manually painted out before filtering the image. Using the polynomial fit, we extract the curvature discontinuity $\kappa(0) = a/(1+a^2)^{3/2}$. The x-component of the center of mass, x^*, is approximated by the average over all x-coordinates of the binarized image of the yarn.

The results of these fits for all yarn types used in this study are reported in Supplementary Table 1.

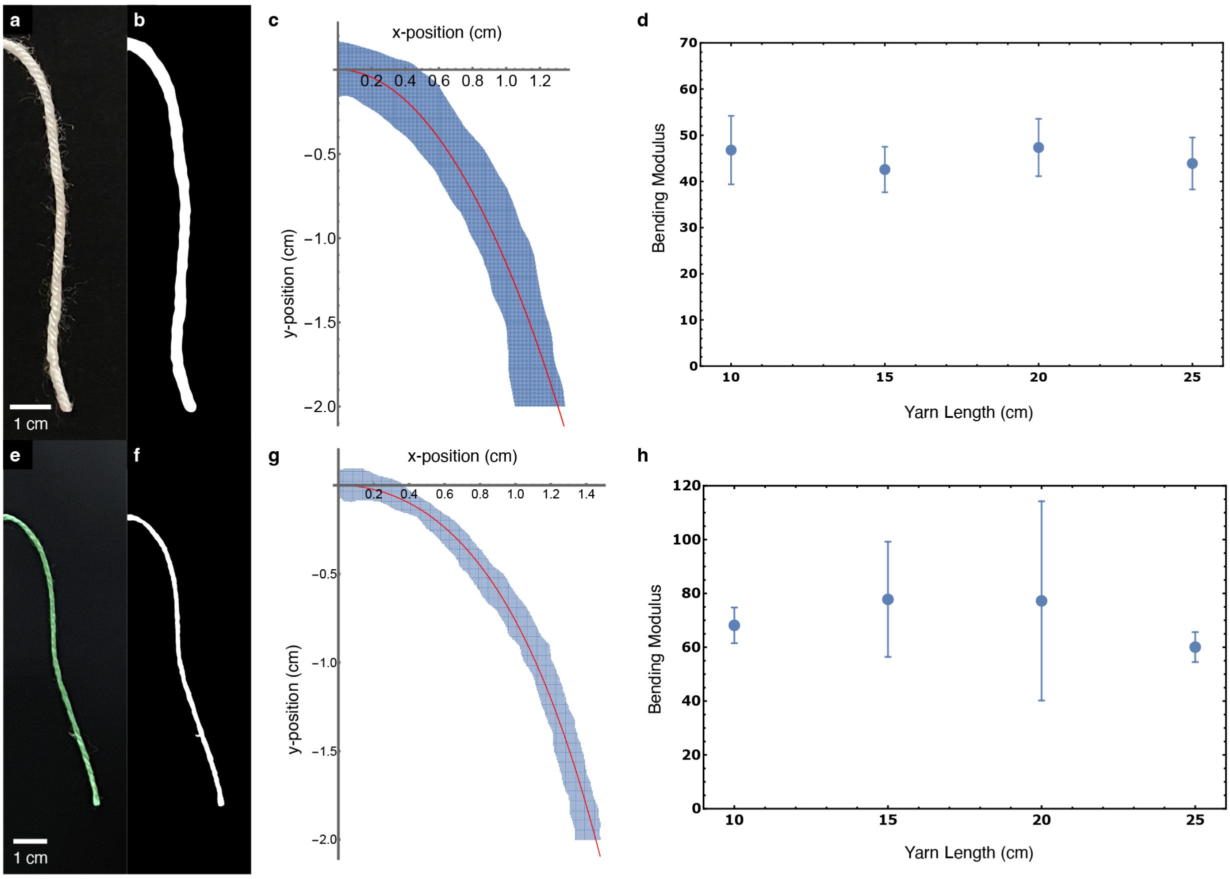

Supplementary Fig. 4. Depiction of the method used to determine yarn bending rigidity. The acrylic yarn bending procedure and results are shown in (**a-d**) while the cotton yarn in (**e-h**). (**a,e**) A segment of yarn is allowed to hang off of a table, with the left end held in place by double sided tape (not shown). The image of the hanging yarn's shape is then binarized with a chosen cutoff intensity to yield the region shown in (**b,f**). The curvature near the taped end is found by fitting a quartic polynomial to the binarized image, with (**c,g**) showing a close-up view of the clamped end. (**d,h**) shows the mean and standard deviation of the measured bending modulus for yarn samples of lengths 10 mm - 25 mm.

SUPPLEMENTARY NOTE 3. Measuring the yarn compressibility and effective interaction potential

Using force vs. probe height compression measurements from the yarn compression experiments (see Supplementary Fig. 5), we find an effective yarn interaction potential energy for use in the simulations. The yarn compression data show nonlinear behavior of the yarn's resisting force as a function of probe height, exhibiting a soft regime for low compression that stiffens as the constituent fibers are forced to pack into a small volume for high compression. Noting that the stress versus strain measurements of the fabric attain maximum stresses of approximately 1 N/mm, we argue that it is sufficient to find an approximate force versus compression depth that follows the yarn compression data up to 1 N. We fit the compression

Yarn Type	B (mN mm^2)
Acrylic Yarn	45.15 ± 5.96
Cotton Yarn	70.8 ± 21.4
Wool blend (glove)	32.80 ± 4.24
Lace-Weight Alpaca Mohair	6.44 ± 2.04
Lace-Weight Blue Mohair	4.89 ± 1.33
Lace-Weight Cashmere	4.34 ± 1.07
Lace-Weight Bamboo	3.48 ± 0.78
Lace-Weight Acrylic	3.72 ± 0.81

Supplementary Table 1. List of yarn bending moduli obtained from cantilever experiments for all yarns used in this study.

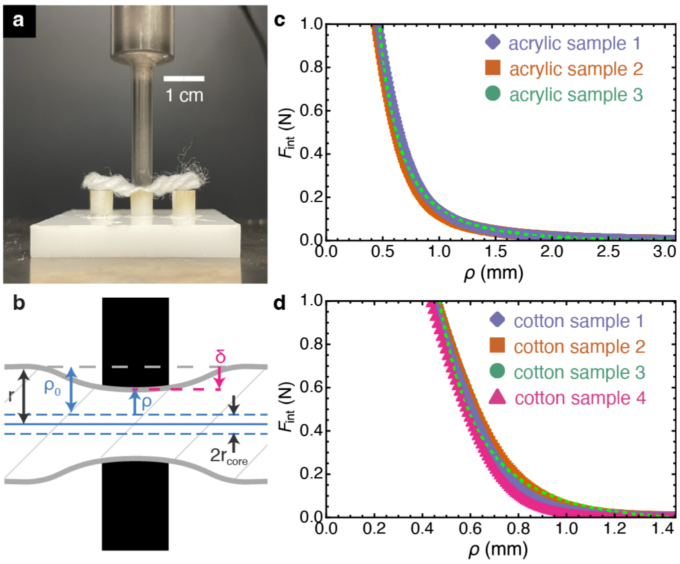

Supplementary Fig. 5. Depiction of the method used to determine the restoring force of the yarn under compression. (**a**) shows the experimental setup with the UTM probe pressing down on yarn that is supported by three rigid pillars. The center pillar has width equal to the probe diameter to best approximate the symmetric deformation illustrated in (**b**). (**c**) and (**d**) show the measured restoring force as a function of probe-to-midline distance ρ for acrylic and cotton samples, respectively. The dashed curve is a fit of the data to the assumed function form given in Supplementary Equation 5.

data to a model force law given by

$$F_{\text{probe}}(\rho) = A_{\text{comp}} k \frac{\rho_0}{p} \left[\left(\frac{\rho_0}{\rho} \right)^p - 1 \right] \qquad \text{(Supplementary Equation 5)}$$

for forces between 10^{-3} N and 1 N, where the lower bound was chosen to cut out fluctuations on the measured force presumably due to the corona of wispy fibers sticking out of the yarn. Note that this form is similar to the contact interaction assumed by Kaldor 2008 [3], except that the exponent p is left as a fitting parameter. Here, $\rho = \delta_c - \delta$ is the thickness of the yarn when the probe is at depth δ, where δ_c is a cutoff depth, modeling an

effective incompressible "core" of the yarn. We use the probe depth at 3 N as the cutoff height. This model was chosen because it captures the compression-stiffening behavior of the yarn for low to moderate compression, where $p > 1$ is a fitting parameter that encodes this nonlinear behavior. Furthermore, $F_{\text{probe}} \to 0$ as $\rho \to \rho_0$, where $\rho_0 = \delta_c - \delta_0$ is the uncompressed thickness of the yarn, where δ_0 is the probe depth at the edge of the yarn (here taken to be when $F_{\text{probe}} \approx 10^{-3}$ N). The fitting parameter k sets the scale of the yarn's compressional rigidity per area, with A_{comp} representing the compressed area of the yarn, which we approximate as the diameter of the UTM tool (5 mm) times the diameter of the yarn being compressed. For small compressions, where $\rho - \rho_0 = \Delta\rho \ll \rho_0$, the compression force is approximately $F_{\text{probe}} \simeq -A_{\text{comp}} k \Delta\rho$ and $A_{\text{comp}} k$ measures an effective spring constant. The results of these fits are shown in Supplementary Table 4.

In the simulations, two yarn segments in contact mutually compress each other. We approximate the compression in terms of the centerline-to-centerline distance of two yarn segments, $R(s, s') \equiv |\boldsymbol{\gamma}(s) - \boldsymbol{\gamma}(s')|$, where $\boldsymbol{\gamma}(s)$ and $\boldsymbol{\gamma}(s')$ are two centerline points. For fixed centerline points, the compressed thickness is taken to be $\rho = R - 2r_{\text{core}}$, where r_{core} is an effective "core radius" of the yarn, representing the hard core cutoff radius, and $\rho_0 = 2r - 2r_{\text{core}}$, where r is the outer radius of the yarn. This type of soft-shell, hard-core model has been used previously in the simulation method of Sperl 2022 [4]. The interaction potential energy density is given by

$$\mathcal{V}_{\text{int}}(\zeta) = \begin{cases} k\frac{(2r-2r_{\text{core}})^2}{p(p-1)}\left[\zeta^{1-p} - 1 - (p-1)(1-\zeta)\right] & \text{for } \zeta < 1 \\ 0 & \text{for } \zeta \geq 1 \end{cases},$$

(Supplementary Equation 6)

where $\zeta \equiv (R - 2r_{\text{core}})/(2r - 2r_{\text{core}})$ is a non-dimensional measure of compressed thickness.

For a more physically accurate model of compression in the future, we would like to study compression in the method that Park and Oh [5] developed for bending, which takes into account the hierarchical structure of spun yarn.

**Method**	**Measurement**	**Stockinette**	**Garter**	**Rib**	**Seed**
Machine Knit	Yarn per stitch (mm)	11.28 ± 0.62	10.55 ± 0.27	16.10 ± 1.58	15.67 ± 0.75
	Yarn diameter (mm)	1.47 ± 0.11	2.07 ± 0.10	2.35 ± 0.15	2.39 ± 0.12
Hand Knit	Yarn per stitch (mm)	17.85 ± 0.95	18.07 ± 0.81	18.32 ± 0.95	18.33 ± 1.15
	Yarn diameter (mm)	2.16 ± 0.15	2.42 ± 0.15	2.49 ± 0.25	2.78 ± 0.32

Supplementary Table 2. The average yarn per stitch and yarn diameter within the stitches for the four types of fabrics made with acrylic yarn. The diameters were measured while the fabrics were in their relaxed (force-free) state. We created samples both by hand and with the knitting machine and note the significant changes in the range of values between the two methods.

**Measurement**	**Stockinette**	**Garter**	**Rib**	**Seed**
Yarn per stitch (mm)	12.28 ± 0.59	16.05 ± 0.54	17.94 ± 0.40	17.32 ± 1.10
Yarn diameter (mm)	1.31 ± 0.11	1.59 ± 0.22	1.38 ± 0.08	1.49 ± 0.14

Supplementary Table 3. The average yarn per stitch and relaxed yarn diameter within the stitches for the four types of fabrics made with the cotton yarn. The diameters were measured while the fabrics were in their relaxed (force-free) state. All of the samples were made using the knitting machine.

	B (mN mm^2)	k (mN mm^{-2})	p
Acrylic yarn (3 samples)	45.15 ± 5.96	0.62 ± 0.10	2.42 ± 0.02
Cotton yarn (4 samples)	70.8 ± 21.4	11.49 ± 3.00	2.94 ± 0.11

Supplementary Table 4. List of yarn bending moduli (B), obtained from cantilever experiments, and compression model parameters (k and p), obtained by fitting to UTM data.

**Yarn Type**	**Stockinette**	**Garter**	**Rib**	**Seed**
Acrylic Yarn	7.74	7.98	8.50	10.70
Cotton Yarn	7.49	7.00	8.81	11.81

Supplementary Table 5. The average area per stitch (in mm^2) for the four types of fabrics made with acrylic and cotton yarn. The caliper used to measure the stitch areas had a measurement precision of 0.01 mm.

SUPPLEMENTARY NOTE 4. Simulation method

4.1. General Methodology

There have been a number of prior studies on yarn-level mechanics of knit stitches, including full 3D continuum elasticity models of yarn [6, 7], as well as reduced-order models [8, 9]. Our simulation method was developed to examine stitch mechanics in a way that retains sufficient detail to explore the impact of stitch geometry (including clasp geometry in the entangled regions, as well as yarn sliding effects), while involving a coarse set of yarn properties (e.g., bending modulus, resistance to compression) to enable a materials-agnostic study. Considering knit stitches as elastica – a continuous curve with bending energy – is a well-established method to consider knit fabric geometry [3, 10–13]. Elastica methods are the middle ground between full three-dimensional continuum elastic models (FEA of the yarn itself) [14, 15] and simplified bead-spring models [16, 17], originally designed for molecular dynamics of polymers and a method that imposes a non-realistic contact geometry between clasped yarns.

We approximated the yarn as an arclength-parametrized space curve $\boldsymbol{\gamma}(s)$ embedded in Euclidean $\mathbb{R}^3$. Equilibrium configurations of the yarn balance stresses due to the (i) bending rigidity of the yarn, (ii) contact interactions of the yarn against itself, and (iii) external, or applied, forces. To this end, we modeled the yarn as inextensible elastica with an interaction

Yarn Type	Measurement	Stockinette	Garter	Rib	Seed
Lace-Weight Alpaca Mohair	Yarn per stitch (mm)	6.93 ± 0.35	6.47 ± 0.33	7.40 ± 0.38	7.40 ± 0.38
	Stitch Area (mm^2)	1.78	1.68	1.61	2.15
Lace-Weight Blue Mohair	Yarn per stitch (mm)	6.73 ± 0.09	6.28 ± 0.08	7.40 ± 0.10	7.62 ± 0.10
	Stitch Area (mm^2)	1.87	1.77	1.60	2.15
Lace-Weight Cashmere	Yarn per stitch (mm)	6.03 ± 0.32	6.03 ± 0.32	6.82 ± 0.36	7.08 ± 0.38
	Stitch Area (mm^2)	1.83	1.63	1.72	2.17
Lace-Weight Bamboo	Yarn per stitch (mm)	5.49 ± 0.65	5.99 ± 0.71	6.73 ± 0.80	6.48 ± 0.77
	Stitch Area (mm^2)	1.76	1.70	1.41	1.85
Lace-Weight Acrylic	Yarn per stitch (mm)	6.46 ± 0.62	6.46 ± 0.62	7.27 ± 0.70	7.00 ± 0.67
	Stitch Area (mm^2)	1.88	1.67	1.62	2.28
Wool Blend (glove)	Yarn per stitch (mm)	$9.83^* \pm 0.32$<br>11.70 ± 0.38	11.54 ± 0.38	12.60 ± 0.41	13.17 ± 0.43
	Stitch Area (mm^2)	5.32*<br>7.74	6.05	6.18	8.50

Supplementary Table 6. The average yarn per stitch and stitch area for the lace weight samples and the therapeutic glove prototype samples. The lace weight samples were fabricated with a STOLL Industrial Knitting Machine and the glove samples were hand knit. The glove sample that is starred was made on 2.00 mm knitting needles (US size 0) and all remaining glove sample data was knit on 2.75 mm knitting needles (US size 2). The caliper used to measure the stitch areas had a measurement precision of 0.01 mm.

energy such that the total energy is given by

$$E_{\text{yarn}} = \int_0^L \mathrm{d}s \left\{ \frac{B}{2} \left| \partial_s \hat{\mathbf{t}}(s) \right|^2 + T + V_{\text{int}}[\boldsymbol{\gamma}; s] \right\} \qquad \text{(Supplementary Equation 7)}$$

where the unit tangent vector is given by $\hat{\mathbf{t}}(s) = \partial_s \boldsymbol{\gamma}(s)$, T is a Lagrange multiplier describing an overall tension that maintains the curve at a constant length L, and the interaction energy is given by

$$V_{\text{int}}[\boldsymbol{\gamma}; s] = \frac{1}{2} \int_0^L \mathrm{d}s' \, \mathcal{V}_{\text{int}} \left(|\boldsymbol{\gamma}(s) - \boldsymbol{\gamma}(s')| \right) . \qquad \text{(Supplementary Equation 8)}$$

The interaction energy density $\mathcal{V}_{\text{int}}(R)$, with $R(s,s') = |\boldsymbol{\gamma}(s) - \boldsymbol{\gamma}(s')|$ is derived from the contact force model with $f_{\text{int}} = -\partial\mathcal{V}_{\text{int}}/\partial R$. Note that we must be careful when integrating the total interaction energy to only count interactions with a minimum separation Δs along the arclength of the yarn. This prevents nearby points from adding divergent contributions to the interaction energy. Refer to Supplementary Table 7 for a list of parameters used in the simulations.

In order to handle the complicated geometry of a knit stitch, we decompose the curve $\boldsymbol{\gamma}(s)$ into a sequence of curve segments $\{\boldsymbol{\gamma}_\sigma(s)\}$ with identified endpoints $\boldsymbol{\gamma}_\sigma(s_{\text{end},\sigma}) = \boldsymbol{\gamma}_{\sigma+1}(s_{\text{start},\sigma+1})$.

To numerically minimize the total energy, we represented the curve $\boldsymbol{\gamma}_\sigma(s)$ as a Bézier curve, expanding in the Bernstein polynomial basis, namely

$$\boldsymbol{\gamma}_\sigma\left(s(t)\right) = \sum_{n=0}^{N} \mathbf{k}_{\sigma,n}\beta_n^N(t)\,, \qquad \text{(Supplementary Equation 9)}$$

where $\{\mathbf{k}_{\sigma,n}\}$ are the *control points* of the curve and

$$\beta_n^N(t) \equiv \frac{N!}{n!(N-n)!}t^n(1-t)^{N-n} \qquad \text{(Supplementary Equation 10)}$$

are Bernstein polynomials. The parameter $t \in [0,1]$ is a re-parametrization of the arclength parameter $s \in [s_{\text{start},\sigma}, s_{\text{end},\sigma}]$ for each segment σ of the resulting Bézier spline curve. However, merely requiring the global curve to be continuous allows for kinks to be introduced into the joints between curve segments. In order to generate realistic results, we additionally require that the unit tangent vector $\hat{\mathbf{t}}(s)$ and its derivative $\partial_s\hat{\mathbf{t}}(s) = \kappa(s)\hat{\mathbf{n}}(s)$ are continuous in space, where $\kappa(s)$ is the curvature and $\hat{\mathbf{n}}(s)$ is the unit normal vector at each point along the curve. These joining conditions between curve segments ensure that the global curve lies within the $\mathcal{C}^3$ continuity class, where $\partial_s^3\boldsymbol{\gamma}(s)$ is continuous everywhere along the spline. We chose to represent each curve segment by degree-5 ($N = 5$) Bézier curves, each specified by six control points. The choice of degree-5 Bézier curves simultaneously gives sufficient flexibility for our simulations whilst maintaining a relatively small number of degrees of freedom and ensuring that the simulated curves remain in the $\mathcal{C}^3$ continuity class [18].

Even though this representation allows control over curve smoothness, variations in control parameters give non-local control over curve shape, giving rise to a large number of local energy minima. This is particularly problematic as a curve segment approaches a straight configuration, due to a degeneracy of control point arrangements for a straight curve. This leads to "vertex bunching," a common problem in geometry optimization [19]. To alleviate this problem, we introduced a cost functional that penalizes such configurations, characterized by small amplitude "wiggles" in the curve shape. We incorporated a regularizing energy of the form

$$E_{\text{reg}} = \frac{C_{\text{reg}}}{2}\int_0^L \mathrm{d}s\,|\partial_{ss}\hat{\mathbf{t}}|^2 \qquad \text{(Supplementary Equation 11)}$$

where C_{reg} is a constant that controls the strength of the regularizing energy.

Rather than simulating a finite swatch of knitted fabric with boundaries, we took advantage of the symmetries of an infinite fabric without boundaries. This enabled a reduction in the scale of the simulation to a single stitch. Since the fabric is a rectangular grid, this

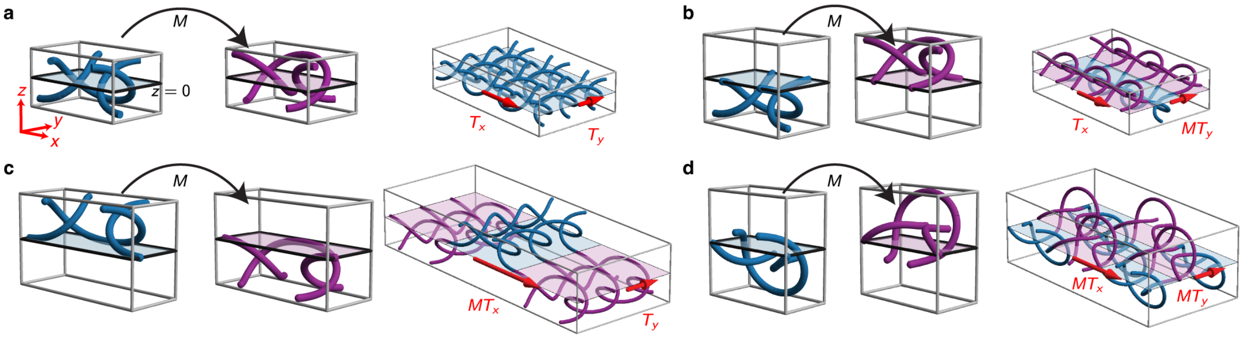

Supplementary Fig. 6. Depictions of the individual stitch cell construction of each of the four fabrics. These diagrams were created using sample outputs of the stitch-level simulations. The yarn radius shown is reduced significantly for clarity. The left-most panels show the geometry of a single relaxed knit stitch contained in a box that outlines the spatial extent of the cell. The center panels show a single relaxed purl stitch, obtained from the knit stitch via the mirror operation M through the $z = 0$ plane, depicted by the colored plane cutting through each of the boxes. The right-most panels show the construction of (**a**) stockinette, (**b**) garter, (**c**) rib, and (**d**) seed fabrics. Each fabric is generated by the repeated action of a combination of mirror operations M, as well as translation operations T_x and T_y, on the knit stitch shown in the left-most panels.

individual stitch cell is a rectangular region of dimensions ℓ_x and ℓ_y (Supplementary Fig. 6), with lattice positions indexed by a pair of integers (m, n), representing the position of a cell relative to a reference cell at $m, n = 0$. The central curve then has a periodic structure given by

$$\boldsymbol{\gamma}_{(m,n)}(s) = M^{f(m,n)} T_y^n T_x^m \boldsymbol{\gamma}_{(0,0)}(s)\,, \qquad \text{(Supplementary Equation 12)}$$

where $T_x : \boldsymbol{\gamma} \mapsto \boldsymbol{\gamma} + \ell_x \hat{\mathbf{x}}$ and $T_y : \boldsymbol{\gamma} \mapsto \boldsymbol{\gamma} + \ell_y \hat{\mathbf{y}}$ are translation operations between stitch cells and $M \equiv (\mathbb{1} - 2\hat{\mathbf{z}} \otimes \hat{\mathbf{z}})$ represents a mirror operation that reflects the stitch path through the midplane of the fabric, converting knits to purls, as depicted in Supplementary Fig. 6. The function $f(m, n)$ sets the number of mirror operations M that are applied at each cell and thus provides information regarding the pattern. It is given by $f(m, n) = 0$ for stockinette fabric, $f(m, n) = n$ for garter fabric, $f(m, n) = m$ for rib fabric, and $f(m, n) = m + n$ for seed fabric. Within a single cell, the path $\boldsymbol{\gamma}_{(0,0)}(s)$ obeys a form of periodic boundary conditions, where the mirror operation may be applied to the unit vector $\hat{\mathbf{t}}(s)$ at each boundary, depending on which stitch pattern being studied. We matched simulations to experiments by setting the length L of the path $\boldsymbol{\gamma}_{(0,0)}(s)$ within a single simulated stitch to the measured yarn length per stitch for each manufactured sample.

We simulated the effect of fabric stretching in the x-direction (y-direction) by numerically minimizing the total yarn energy $E_{\text{yarn}}[\gamma_{(0,0)}]$ at fixed stitch cell dimension ℓ_x (ℓ_y), while allowing the transverse dimension ℓ_y (ℓ_x) to vary. This minimization was performed using the Sequential Least Squares Programming (SLSQP) method in the `scipy.optimize` Python package (`https://docs.scipy.org/doc/scipy/reference/optimize.html`), which is a gradient-free optimization algorithm that allows a number of equality and inequality constraints to be specified; in particular, we fixed the yarn length L to be constant. To generate a 1D energy landscape $E(\ell_\mu)$ (with $\mu = x, y$), we first started with a guess for an initial, un-stretched configuration, at an initial stitch dimension ℓ_μ, and numerically minimized that

configuration. We then incremented or decremented the stitch dimension $\ell_\mu \to \ell_\mu \pm \Delta\ell$ and used the result of the minimization as new initial conditions for minimizing the energy over this new cell dimension; we generated full 1D landscapes using this 0th order parametric continuation, making sure to sweep in both $\pm\Delta\ell$ directions to bracket an energy minimum. Since this minimization approach is prone to finding local, metastable energy minima, we performed this sweep on four different initial stitch configurations, accepting the lowest energy value as the accepted simulation result, in order to search for better approximations to the "true ground state" of the stitch. Simulated annealing methods may get closer to this global minimum. However, we found that the Bézier curve representation suffers from a large number of near-degenerate configurations, complicating the application of simulated annealing methods.

With a given energy landscape $E(\ell_\mu)$, we applied a discrete, midpoint derivative and found the force profile $f_x(\ell_x) = \mathrm{d}E/\mathrm{d}\ell_x$ (or $f_y(\ell_y) = \mathrm{d}E/\mathrm{d}\ell_y$). The completely relaxed, force-free configuration of the stitch corresponds to the point where $f_x = f_y = 0$, which can equivalently be found in either the $E(\ell_x)$ or the $E(\ell_y)$ landscapes since allowing the transverse dimension to vary freely in minimization is equivalent to specifying a zero-force condition on that dimension. The force-free configuration was found with a using 3rd order polynomial interpolation on data where high-energy compression simulations were eliminated. Denoting the stitch cell dimensions of that completely relaxed configuration as $\ell_{x,0}$ and $\ell_{y,0}$, we converted force data to nominal stress via $\sigma_{xx} = f_x/\ell_{y,0}$ and $\sigma_{yy} = f_y/\ell_{x,0}$, expressed as a function of the linear strain components $\varepsilon_{xx} = (\ell_x - \ell_{x,0})/\ell_{x,0}$ and $\varepsilon_{yy} = (\ell_y - \ell_{y,0})/\ell_{y,0}$.

While static friction is expected to play an important role in various aspects of fabric mechanics, our focus on using energy-minimizing configurations of stitches to approximate both the "relaxed" ($f = 0$) and "deformed" ($f \neq 0$) states of the stitches led us to develop simulations that are inherently different than the use of simulated damping from friction (i.e. gradient descent) to "evolve" configurations towards equilibrium states. This simplification allows us to focus on details of yarn geometry in determining equilibrium response, without needing to worry about specifying certain deformation paths. Given that the Bézier representation uses control points that provide non-local control over yarn shape, it is difficult to incorporate damping terms in these simulations; representations that yield local control, such as other B-splines, would be more suited for investigating friction and path-dependence of deformation protocols (e.g. loading vs. unloading). However, we do find that some level of static friction is necessary to reproduce the deformation of seed stitches; we introduce constraints in order to simulate this effect (see next section).

4.2. Restricting sliding with arclength constraints

Our simulation method of minimizing an elastica energy functional over topologically-constrained configurations of yarn does not incorporate effects of friction. Given the wispy, corrugated texture of the yarn, we expect that friction may play an important role in reducing the ability of the yarn to slide against itself in certain configurations. We hypothesize that this effect may be particularly relevant for seed stitch, as their cross-over regions do not clasp as completely as other stitches, which can be seen in Supplementary Fig. 6. In particular, seed possesses relatively straight segments oriented along $\hat{\mathbf{y}}$, even in the un-stretched configuration, allowing for a soft sliding motion that is distinct from the soft near-rigid rotation of odd connecting yarn segments (described in SUPPLEMENTARY NOTE 8).

To demonstrate the effect of contact sliding, we consider the extreme limit of quenched

	B (mN mm^2)	k (mN mm^{-2})	p	r (mm)	r_{core} (mm)	L (mm)	r_{core}/r
Stockinette (acrylic)	46	0.6	2.4	0.74	0.335	11.3	0.453
Garter (acrylic)	46	0.6	2.4	0.54	0.245	10.6	0.454
Rib (acrylic)	46	0.6	2.4	1.18	0.415	16.1	0.352
Seed (acrylic)	46	0.6	2.4	1.20	0.290	15.7	0.242
Stockinette (cotton)	70	11.5	2.9	0.66	0.310	12.28	0.470
Garter (cotton)	70	11.5	2.9	0.80	0.420	16.05	0.525
Rib (cotton)	70	11.5	2.9	0.69	0.405	17.32	0.587
Seed (cotton)	70	11.5	2.9	0.75	0.430	17.94	0.573

Supplementary Table 7. List of yarn material parameters used in simulations. We adjusted the hard-core radius r_{core} to obtain better agreement with the stress-vs-strain curves obtained from experiments. For the acrylic yarn, the core radius averages 37.5% of the yarn radius with a standard deviation of ±10.1%. For the cotton yarn, the core radius averages 53.9% of the yarn radius with a standard deviation of ±5.3%. This suggests the core radius has a dependence on the yarn type and is slightly influenced by the fabric type. How the fabric manufacturing process for different fabric types affects the core radius is currently unknown. Generally, increasing the core radius in the simulation leads to a stiffer fabric for all four fabric types, but the exact dependency of the constitutive model on the core radius is a subject of further study. Existing simulations looking to replicate experimental stretching responses often have many more fitting parameters with complex optimization schemes [4].

sliding. This is implemented in the energy minimization through as pair of constraints that break the reptation symmetry of the yarn. Using our decomposition of yarn into cross-overs and connections, we can approximate the arclength coordinate of the ith contact point, s_i, as “half-way” between the ends of the yarn in the corresponding cross-over region, which are at points $s_{i,0}$ and $s_{i,1}$, taking $s_i = (s_{i,0} + s_{i,1})/2$. The slide-quenching constraints amount to ensuring that the total arclength of yarn joining two neighboring cross-over regions remains constant under deformation, i.e. $s_{i+1} - s_i = s^0_{i+1} - s^0_i$, where s^0_i are the corresponding arclength positions in the un-stretched state. Within each stitch there are four such contact points, so in principle there need to be four such constraints. However, the mirror symmetry of the stitch about the yz-plane passing through its middle relates two of the arclength coordinates, and the total arclength constraint implies that $\sum_i s_i = L$, leaving only two constraints that need to be enforced, namely

$$\begin{aligned} &s_2 - s_1 = s^0_2 - s^0_1 \text{ and} \\ &s_3 - s_2 = s^0_3 - s^0_2\ . \end{aligned} \qquad \text{(Supplementary Equation 13)}$$

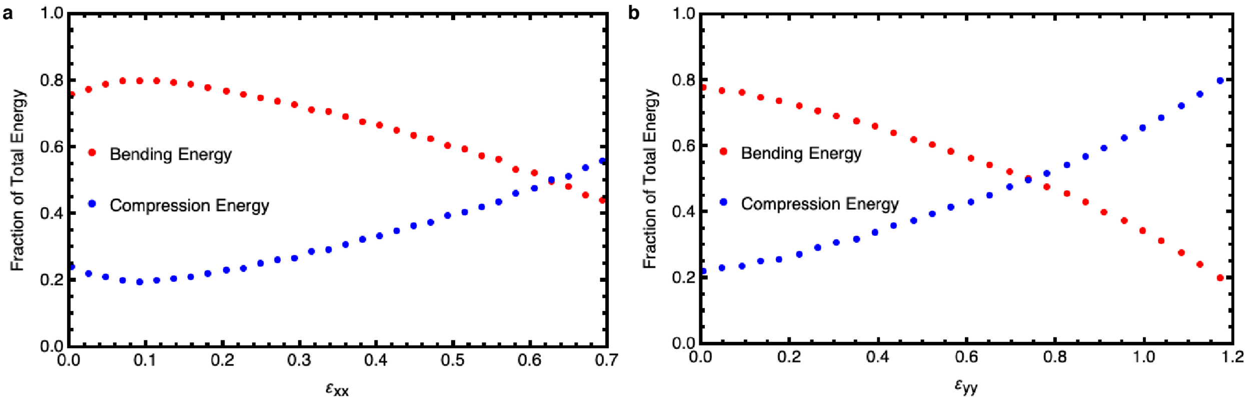

Supplementary Fig. 7. Energy breakdown for cotton garter sample stretched in the x- (**a**) and y-directions (**b**). The compression (blue) and bending (red) energies are given as percentages of the total energy at each value of strain.

Note that the addition of these constraints require that stretched stitches inherit information about the un-stretched equilibrium, namely $\{s_i^0\}$. This dependence on a reference configuration distinguishes the elasticity of slide-quenched fabrics from those that allow for sliding in a way reminiscent to the difference between elastomeric materials (e.g. polymer gels and rubbers) that attain rigidity via permanent cross-links, versus so-called "topological" constraints. We leave further explorations of quenched versus annealed sliding for future studies.

4.3. Simulation energy analysis

Some of the value in the yarn-level simulations is the ability to determine the components of the energy of the yarn as given in Supplementary Equation 7. For each simulation, we can extract the components of energy due to yarn bending and yarn compression. Generally, as seen in Supplementary Fig. 7 with the cotton garter sample, the bending energy dominates at low strain. As the strain increases, the proportion of compression energy increases, finally overcoming the bending contribution at high enough strain. This transition is typically smooth, though an exception to this is discussed in SUPPLEMENTARY NOTE 4.5, for acrylic seed. Values for the total energy per stitch and energy ratios for all fabric types and both yarn types can be found in Supplementary Table 8. This simulation data supports the Reduced Symmetry model, where bending is the dominant energy contribution at low strain.

4.4. Observed "jamming" response in low-stress regime

At very low stresses, we sometimes observe an initial high-rigidity response before the fabric softens into a linear stress-strain response. This behavior is seen in both experimental (see acrylic garter pulled in the x-direction and rib in the y-direction (Supplementary Fig. 3a), cotton stockinette and garter pulled in the x-direction (Supplementary Fig. 3b), and the therapeutic glove test samples for stockinette in the x-direction and stockinette and rib in the y-direction (Supplementary Fig. 3c)) and simulation (see stockinette and seed in

	E_{total} (J)	$E_{\text{compression}}/E_{\text{total}}$	$E_{\text{bending}}/E_{\text{total}}$
Stockinette (acrylic)	0.249	0.130	0.870
Garter (acrylic)	0.175	0.026	0.974
Rib (acrylic)	0.189	0.391	0.609
Seed (acrylic)	0.288	0.459	0.541
Stockinette (cotton)	0.401	0.233	0.767
Garter (cotton)	0.254	0.216	0.784
Rib (cotton)	0.139	0.044	0.956
Seed (cotton)	0.262	0.172	0.828

Supplementary Table 8. List of total energy per stitch, the ratio of compression energy to total energy, and the ratio of bending energy to total energy as given by zero-force simulations. On average over all fabric types in both yarn types, the bending energy is 79% ± 15% of the total energy.

the x-direction and seed in the y-direction in Supplementary Fig. 2) data. Other groups studying knits made with incompressible yarn have seen similar low-stress, high-rigidity regions [20]. Postle [21] described this behavior as a jammed regime where forces normal to the stretching direction prevent the yarn from rearranging and the fabric from extending. Since jamming is a contact-dependent phenomenon, jamming behavior present in simulation results that is not found in experiments may be a result of our contact model.

4.5. Instability in acrylic seed

Shown in Fig. 2, the simulation stress-strain curve for acrylic seed differs from the experimental result. Further investigation into the simulation shows a buckling instability at a strain of ≈ 0.8, where the stitches move out of the z-plane to reduce the compression energy as shown in Supplementary Fig. 8. Due to the checkerboard pattern of knit and purl stitches, seed fabric is uniquely able to express this out-of-plane buckling instability in comparison to other fabric types. For hard colloidal spheres, out-of-plane buckling (from two-dimensions to three-dimensions) results in a square lattice [22]. Neighboring particles want to go opposite directions out of the plane, such that a neighboring pair have one particle above the plane and one below. The stitch configuration of seed enables this transition, whereas the stitch configurations of the three other fabric types prevent it. Simulations for other stitch patterns with more limited frustration may also allow this buckling. Seed also has the highest proportion of compression energy of all the fabric types, as seen in Supple-

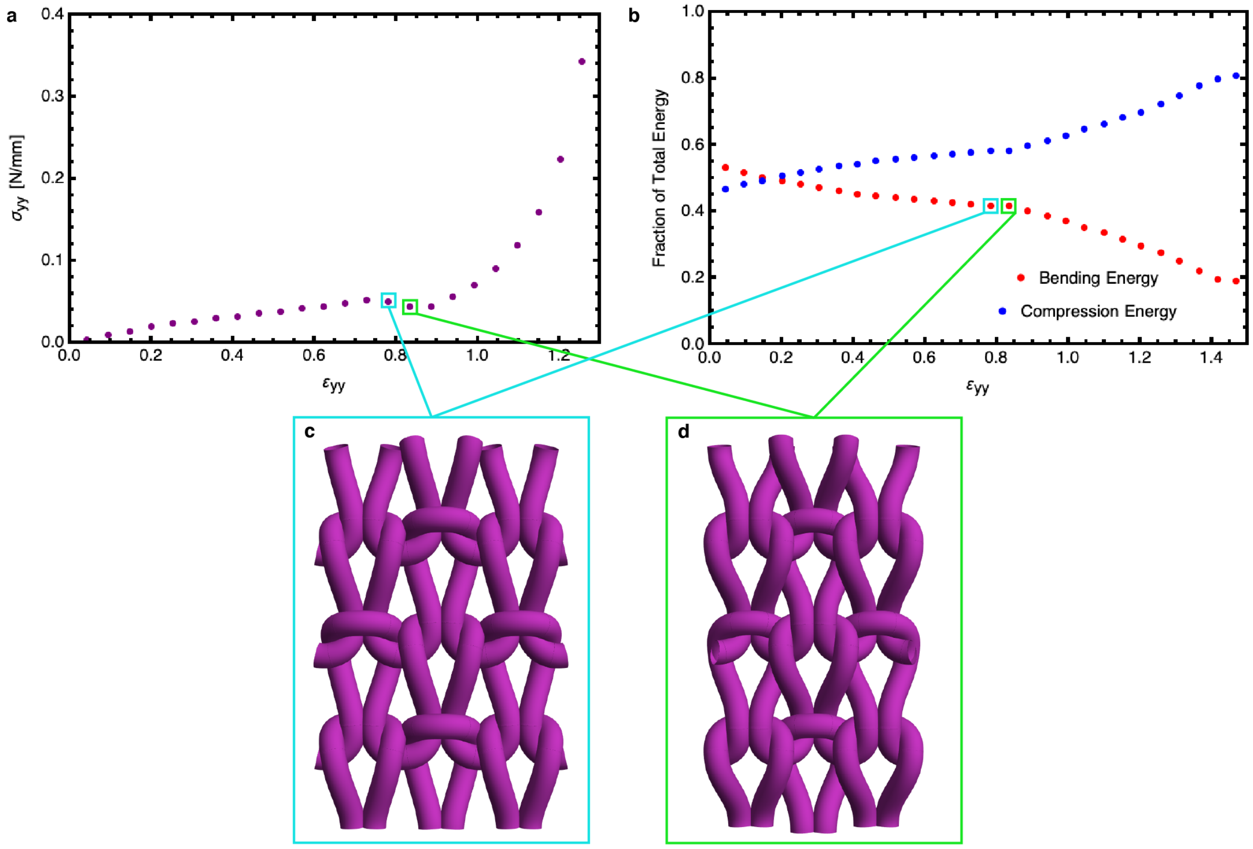

Supplementary Fig. 8. Energy breakdown (**b**) and stitch configurations (**c**, **d**) for the buckling instability in seed acrylic simulations that correlate to the abnormality in the stress-strain plot (**a**). The stitch configurations show a tiling of 3 by 3 stitches in the $x-y$ plane before (**c**) and after (**d**) the buckling instability occurs. The buckling is characterized by sudden overlaps of the entangled regions of neighboring stitches. These strain locations in the energy breakdown in **b** show that the onset of buckling is correlated with a decrease in the relative compression energy while the relative bending energy increases, counter to the general trends seen for each component of the energy. These renderings (**c,d**) were made using the outputs of the seed simulations.

mentary Table 8, which would make it more susceptible to contact-induced buckling. As seen in Supplementary Table 10, the Poisson ratio ν_{xy} for the simulation is approximately four times larger than experiment. This larger Poisson ratio is a marker of the simulation buckling, but our current analysis is insufficient to determine causality. The non-monotonic behavior of the contact energy in Supplementary Fig. 8b indicates that there is an instability in the numerics caused by the contact model.

4.6. Accounting for Manufacturing Tension

In the simulation, manufacturing tension is relevant during initialization of the fabric before it is stretched or deformed in any way. Others have simulated. the actual knitting process [20], implemented a shrinking factor that reduces the arc-length of segments of yarn until the fabric settles into a rest-state [3], or taken a picture of a physical sample and used

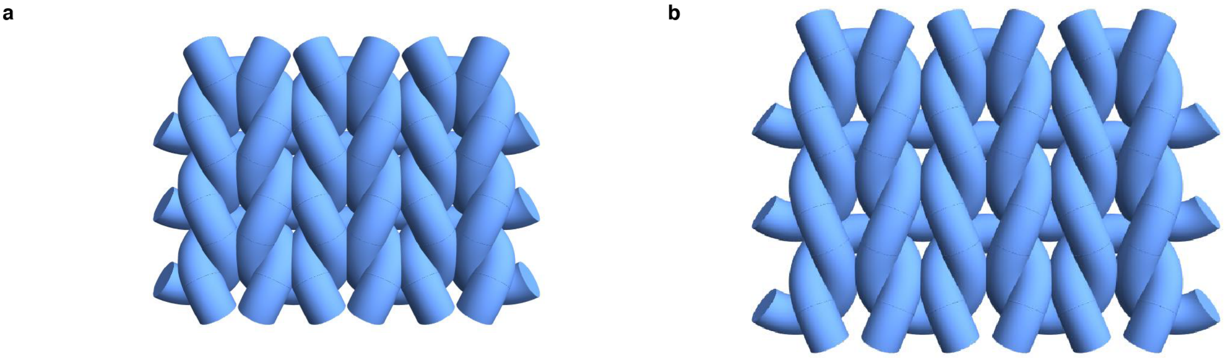

Supplementary Fig. 9. Stitch configurations of simulation outputs of stockinette fabric with different length of yarn per stitch constraints, **(a)** 10.5 mm and **(b)** 12.5 mm, to represent different levels of manufacturing tension. These stitch configurations are made from multiple tilings of the stitch unit cell to create a fabric that is three stitches wide by three stitches high.

that geometry as an input of the simulation that is then relaxed to near force-balance [4]. Many don't consider tension at all [23]. Of these initialization strategies, the method we use is closest to that of Sperl et al. [4]; we start with a input geometry inspired by the actual geometry of the stitches within the fabric. We then impose constraints and yarn properties. The length constraint, which fixes the length of yarn per stitch, is how we control how tightly the stitches are manufactured. Once these input properties are imposed, we allow the simulation to find the minimum energy configuration that fulfills these constraints. In Supplementary Fig. 9, we show how we can control the tightness of the stitches by changing the length of yarn per stitch.

We also consider how tension may affect the yarn structure by allowing the core radius of the yarn to vary. In this way, we account for how the yarn may change its compressibility under tension without a physical model for that phenomenon, which is currently poorly understood. We also take measurements of the yarn radius *in situ* to represent changes in yarn radius under tension. Worsted weight yarn, such as the acrylic and cotton yarns used in our samples, often visibly change radius under tension.

4.7. Friction

Friction is often included in dynamic simulations of knitted fabrics [3, 8, 20]. We do not include friction in our stitch-level simulations, primarily because our simulations are static. Friction can not be incorporated into an energy minimization scheme. Including a dissipative term such as friction into a static simulation is not supported by both the general simulation method and the specific way we minimize the energy.

For each set of stitch cell dimensions, we iteratively change the shape of the stitch to find the minimum energy configuration [24], as previously described. We are not continuously stretching the stitch cell. Each set of stitch cell dimensions is a single simulation, unconnected to other simulations of different stitch cell dimensions. Static simulations of this kind well suit our purposes to use simulations to investigate the role of topology on fabric mechanics. By finding the mechanical equilibrium point of the stitch cell for each set of given dimensions, we well represent the mechanics of our experiments. Our stress-strain

simulation results are able to replicate the shape of both the linear and non-linear elastic responses of knit fabrics, which has yet to be achieved for knits made of compressible yarns.

As mentioned in Supplementary Note 4.1, we use the Sequential Least Squares Programming (SLSQP) method in the `scipy.optimize` Python package (https://docs.scipy.org/doc/scipy/reference/optimize.html) to conduct our energy minimization. This method of optimization does not use gradients and cannot incorporate a dissapative energy term. We chose this method due to its suitability for our specific static simulations; this minimization method is able to take large steps in the energy landscape to converge faster and can often recover from divergent energy configurations. To include a dissapative energy term like friction, we would have to move to a dynamic simulation and use a different optimization scheme, such as gradient descent.

Prior research on rib fabric made of incompressible yarn shows that energy lost to friction is very small, at most the totalling the energy of the third-largest contribution for the entirety of the knit's elastic response [20]. Since we are using a compressible yarn with an appreciable energy contribution from yarn compression (see Supplementary Fig. 7) and stretching fabric in the quasi-static regime, we estimate that friction has a similarly small, if not smaller, contribution to our fabric.

We do not see frictional effects on the elasticity of the knit fabrics when we iteratively repeat extension experiments, as seen in Supplementary Fig. 3. This lack of measurable frictional effect on the experimental samples indicates that friction must be a very small contribution. This is supported by the fact that we can wear clothes multiple times without them losing their elasticity. Socks in particular retain their elasticity over multiple wears even though they are constantly being stretched and deformed with every step. If friction had a large role in knit fabric mechanics, socks would become single-use items.

SUPPLEMENTARY NOTE 5. Transverse stress-strain behavior

In the uniaxial stretching experiments, the fabrics attain an hourglass-like waist as the force acting along one direction causes a response in the transverse direction. This response is a result of the fabric's Poisson effect, i.e. stretching the fabric in one direction thins it in transverse directions. For example, if the fabric is stretched in the x-direction so that the strain component ε_{xx} is positive, then it thins in the y-direction. Due to the constraints on transverse deformation imposed by clamps on two of the edges, the strain field component $\varepsilon_{yy} < 0$ varies along the fabric, and the magnitude of the strain reaches a maximum where the waist narrows. The pin tracking approach we employed measures the deformation in this region.

Due to the anisotropy of each fabric, the transverse deformation is characterized by two Poisson ratios, ν_{yx} and ν_{xy}. In principle, these ratios describe the linear response of the fabric under two different deformation protocols: if the fabric is stretched in the x-direction (i.e. $\varepsilon_{xx} > 0$) then the transverse response is $\varepsilon_{yy} = -\nu_{yx}\varepsilon_{xx}$; if the fabric is stretched in the y-direction then the transverse response is $\varepsilon_{xx} = -\nu_{xy}\varepsilon_{yy}$. We measured these ratios for experimental and simulation results, and they are reported in Supplementary Table 10, Supplementary Table 12 and Supplementary Table 20.

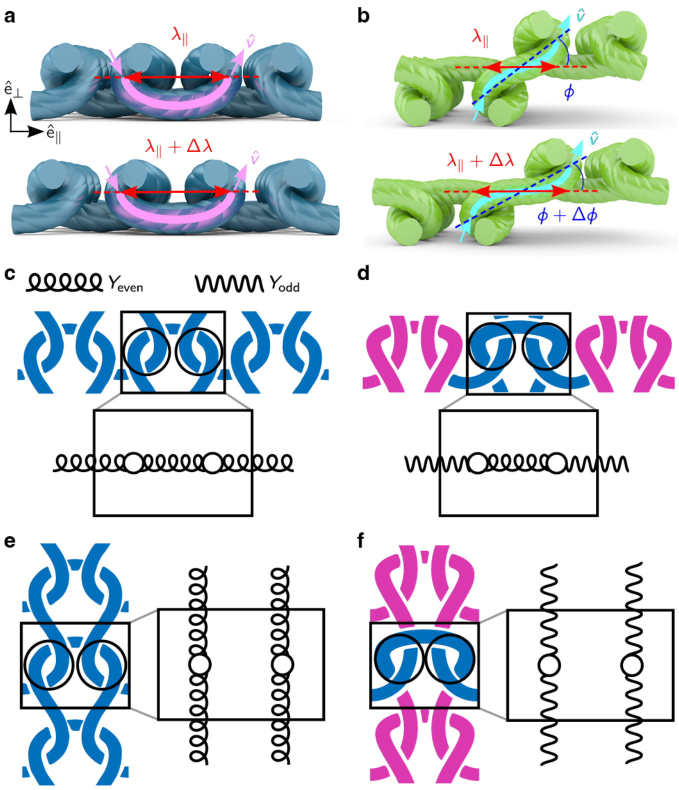

Supplementary Fig. 10. (**a**,**b**) Diagrams of the even (**a**) and odd (**b**) regions of stockinette and rib, respectively, with labels for the re-parameterized geometric variables used in SUPPLEMENTARY NOTE 8. These renderings we done using sample outputs of the stitch-level simulations. For (**a**, **b**), horizontal is the x-direction of the fabric and into-the-plane is the y-direction. (**c**, **d**, **e**, **f**) Effective spring network elements for like-stitch (**K**-**K** or **P**-**P**) neighbors in the x-direction (**c**), unlike-stitch (**K**-**P**) neighbors in the x-direction (**d**), like-stitch neighbors in the y-direction (**e**), and unlike-stitch neighbors in the y-direction (**f**). For (**c**, **d**, **e**, **f**), horizontal is the x-direction of the fabric and vertical is the y-direction. Circled areas indicate entangled regions.

SUPPLEMENTARY NOTE 6. Nonlinear constitutive model

Here, we provide a scaling rationale for the constitutive relationship. The results presented here are consistent with the RS model of SUPPLEMENTARY NOTE 8, including the form of the strain-stiffening term. First, consider the various lengthscales that describe a knit stitch. These include the yarn radius r, the length of yarn per stitch L, and a "bending lengthscale" $\ell_\mathrm{b} \sim \sqrt{B/T}$, where B is the yarn's bending modulus and T is the tension of the yarn, which is obtained from dimensional analysis. In fact, ℓ_b has a simple physical interpretation: if one considers an arc of radius R, the work done in stretching the arc's radius to $R+\delta R$ under constant tension scales as $\sim T\delta R$, which is counteracted by a change in the bending energy, which scales as $\sim -B\delta R/R^2$, and the two generalized forces are in equilibrium if $R \sim \ell_\mathrm{b} \sim \sqrt{B/T}$. Thus, ℓ_b can be regarded as the radius of curvature that dominates the bending energy of a curve in mechanical equilibrium under tension. As T increases, ℓ_b decreases, so that the bending energy of a stitch is increasingly concentrated to small regions of high curvature, which must be located at the entangled regions. Note

that in the arc approximation, the ℓ_{b} is both the radius of curvature and the arclength of the curved regions, so that $E_{\mathrm{bend}} \sim 1/\ell_{\mathrm{b}}$. Since the radius r of the yarn represents a lower limit of the radius of curvature and the stitch length L determines the periodicity of yarn shape, ℓ_{b} is bounded by these two lengths: $r \lesssim \ell_{\mathrm{b}} \lesssim L$.

The limits of this region correspond to two physical cases. Case one, $\ell_{\mathrm{b}} \approx L$, occurs when there is little to no applied external force. Under small strains ε, the bending length decreases linearly, such that changes in ℓ_{b} go as $\Delta\ell_{\mathrm{b}} \sim -\varepsilon\ell_{\mathrm{b}_0}$, where ℓ_{b_0} is the bending length at zero strain. This results in the linear stress-strain relationship $\sigma_{\mathrm{low}} \propto \varepsilon$. Case two, $\ell_{\mathrm{b}} \approx r$, occurs when the fabric is under high external load. Here, the yarn segments within each crossover region clasp increasingly tightly around each other. In this regime, the total length of yarn L can be approximated as $L \approx c(\lambda_{\mathrm{max}} + r)$, where c is a numerical prefactor of $\mathcal{O}(1)$ and $\lambda_{\mathrm{max}} \approx (1+\varepsilon)\lambda_0$, the maximal separation between crossover regions, varies linearly with the average separation between crossover regions in the unstrained case, λ_0. Therefore, the bending length can be approximated as $\ell_{\mathrm{b}} \approx r \approx (L/c) - \lambda_{\mathrm{max}} \approx A(1-\alpha\varepsilon)$, where A and α are constants determined by the material properties of the yarn. The bending energy scales as $E_{\mathrm{bend}} \sim \ell_{\mathrm{b}}^{-1} \sim (1-\alpha\varepsilon)^{-1}$. This implies that the high-stress regime scales as $\sigma_{\mathrm{high}} \sim \partial E_{\mathrm{bend}}/\partial\varepsilon \sim (1-\alpha\varepsilon)^{-2}$. This is consistent with our elastica analysis in the preceding section, where the high-tension limit $q \gg 1$ recovers the same bending energy scaling form.

While the low-stress regime is determined by topology, the high-stress regime is dominated by the material properties of the yarn. Combining these limiting behaviors leads us to a stress-strain relationship $\sigma(\varepsilon) \approx \sigma_{\mathrm{low}}(\varepsilon) + \sigma_{\mathrm{high}}(\varepsilon)$, which fits our experimental and simulation data quite well, as shown in Fig. 2, Supplementary Fig. 13, and Supplementary Fig. 2.

SUPPLEMENTARY NOTE 7. Uniaxial constitutive model and fitting

Our constitutive model for a sample of knitted fabric under uniaxial stress is given by

$$\begin{cases} \sigma_{xx}(\varepsilon_{xx},\varepsilon_{yy}) = C^0_{xxxx}\varepsilon_{xx} + C^0_{xxyy}\varepsilon_{yy} + \beta_{xx}\left(\frac{1}{(1-\alpha_{xx}\varepsilon_{xx})^2} - 1 - 2\alpha_{xx}\varepsilon_{xx}\right) \\ \sigma_{yy}(\varepsilon_{xx},\varepsilon_{yy}) = C^0_{yyyy}\varepsilon_{yy} + C^0_{yyxx}\varepsilon_{xx} + \beta_{yy}\left(\frac{1}{(1-\alpha_{yy}\varepsilon_{yy})^2} - 1 - 2\alpha_{yy}\varepsilon_{yy}\right) \end{cases},$$

(Supplementary Equation 14)

where C^0_{xxxx}, C^0_{yyyy}, C^0_{xxyy}, and C^0_{yyxx} are components of the rigidity tensor $C_{ijkl} \equiv \partial\sigma_{ij}/\partial\varepsilon_{kl}$, evaluated in the low-strain, linear elastic limit wherein $\sigma_{ij} \approx C^0_{ijkl}\varepsilon_{kl}$. The constants α_{xx} and α_{yy} characterize the finite extensibility of knitted fabric when stretched in orthogonal directions, with β_{xx} and β_{yy} setting the stress scale of the strain-stiffening regime. Our experimental results find significant asymmetry between C^0_{xxyy} and C^0_{yyxx} components, see Supplementary Table 9, Supplementary Table 11, and Supplementary Table 19. This is backed up by simulations showing significant asymmetry between x-strain and y-strain. Therefore, we do not enforce the standard symmetry $C^0_{xxyy} = C^0_{yyxx}$ in our model. We hypothesize that this is due to changes in non-local contact interactions that occur when the fabric is strained in different directions.

We obtained values of these eight parameters for each fabric by fitting the constitutive relations to data via a least-squares scheme. For each fabric, we obtained two independent data series from the uniaxial stretching experiments: (i) measured triplets $S_x = \left\{\left(\varepsilon^{\mathrm{data}}_{xx}, \varepsilon^{\mathrm{data}}_{yy}, \sigma^{\mathrm{data}}_{xx}\right)\right\}$ of strain and stress in the x-direction and (ii) measured triplets $S_y =$

$\{(\varepsilon_{yy}^{\text{data}}, \varepsilon_{xx}^{\text{data}}, \sigma_{yy}^{\text{data}})\}$ of strain and stress in the y-direction. We then minimize the functional

$$I = \sum_{(\varepsilon_{xx}^{\text{data}}, \varepsilon_{yy}^{\text{data}}, \sigma_{xx}^{\text{data}}) \in S_x} \left(\sigma_{xx}(\varepsilon_{xx}^{\text{data}}, \varepsilon_{yy}^{\text{data}}) - \sigma_{xx}^{\text{data}}\right)^2 + \sum_{(\varepsilon_{yy}^{\text{data}}, \varepsilon_{xx}^{\text{data}}, \sigma_{yy}^{\text{data}}) \in S_y} \left(\sigma_{yy}(\varepsilon_{xx}^{\text{data}}, \varepsilon_{yy}^{\text{data}}) - \sigma_{yy}^{\text{data}}\right)^2$$

(Supplementary Equation 15)

with respect to the seven unknown parameters in the constitutive relation. However, minimizing this functional alone is insufficient because it ignores constraints imposed by the boundary conditions of the fabric. The stress-free boundary conditions couple longitudinal and transverse strains, giving rise to the Poisson effect. In order to introduce this coupling when fitting the data, we determine the pair of Poisson ratios, ν_{yx} and ν_{xy}, via linear fits to data sets S_x and S_y, respectively. Then we minimize the functional I under the constraints that the linear rigidity tensor components are consistent with these Poisson ratios via the relationships $\nu_{yx} = C^0_{yyxx}/C^0_{yyyy}$ and $\nu_{xy} = C^0_{xxyy}/C^0_{xxxx}$. Values obtained for the fitting parameters are shown in Supplementary Table 9, Supplementary Table 11, and Supplementary Table 19. The Young's moduli are then given by

$$\begin{aligned} Y_x &= C^0_{xxxx} - \frac{C^0_{xxyy}C^0_{yyxx}}{C^0_{yyyy}} = (1 - \nu_{xy}\nu_{yx})C^0_{xxxx} \\ Y_y &= C^0_{yyyy} - \frac{C^0_{yyxx}C^0_{xxyy}}{C^0_{xxxx}} = (1 - \nu_{yx}\nu_{xy})C^0_{yyyy} \end{aligned}$$

(Supplementary Equation 16)

where we see that $Y_x/Y_y = C^0_{xxxx}/C^0_{yyyy}$, yet $Y_x \neq C^0_{xxxx}$ and $Y_y \neq C^0_{yyyy}$. Values of the Young's moduli, along with error estimates based on the variance obtained from least squares fitting, are shown in Supplementary Table 10 and Supplementary Table 12. These figures are plotted in Supplementary Fig. 11.

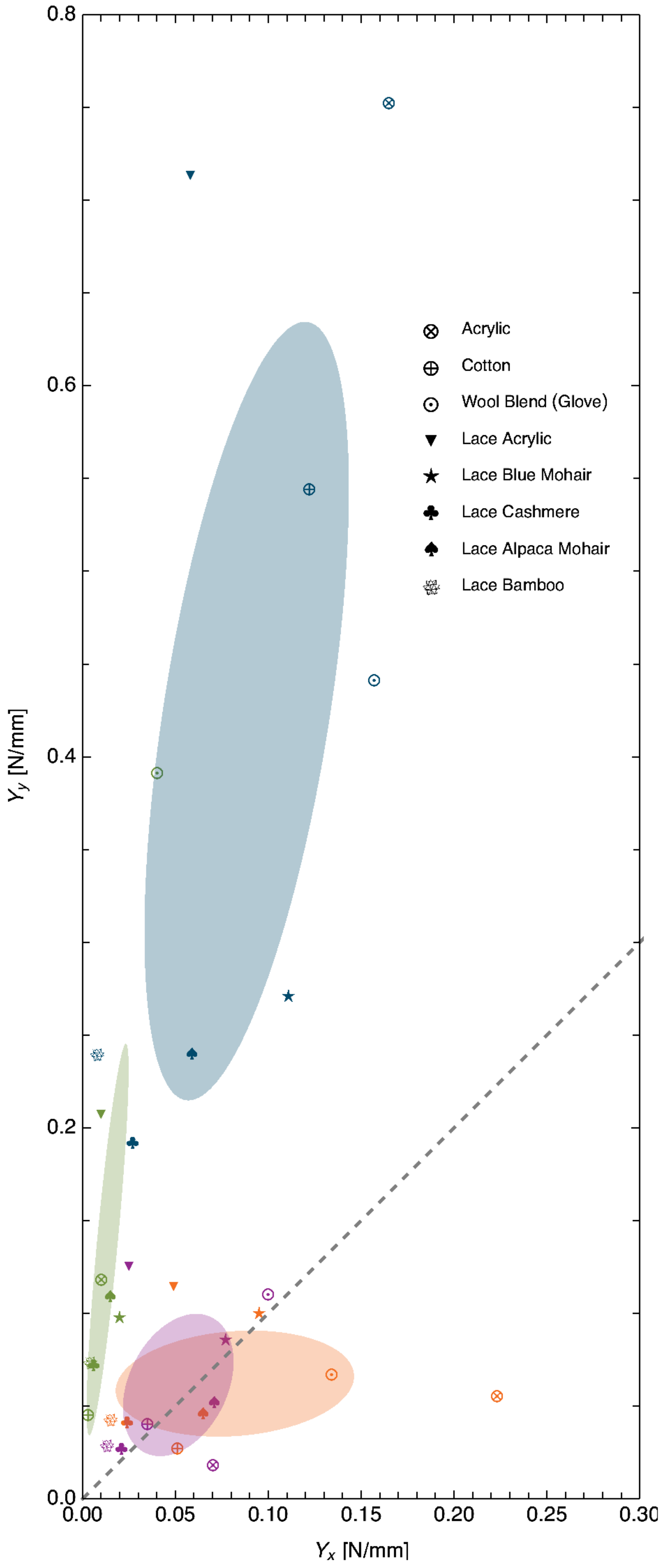

Supplementary Fig. 11. Rigidity-rigidity plot for all fabric samples, where Y_i is the Young's modulus in the ith direction. The colored ellipses represent one standard deviation for each of the four fabric types and are oriented along the principal axes: stockinette in blue, garter in orange, rib in green, and seed in purple. The gray dashed line represents a isotropic mechanical response.

	C^0_{xxxx} (N/mm)	C^0_{yyyy} (N/mm)	C^0_{xxyy} (N/mm)	C^0_{yyxx} (N/mm)	α_{xx}	α_{yy}	β_{xx} (N/mm)	β_{yy} (N/mm)
Stockinette (experiment)	0.204	0.930	0.088	0.413	1.111	2.537	0.046	0.010
Stockinette (simulation)	0.200	0.753	0.040	0.341	1.391	2.971	0.005	0.013
Garter (experiment)	0.241	0.060	0.036	0.029	1.170	0.802	0.022	0.022
Garter (simulation)	0.252	0.038	0.103	0.019	1.073	1.135	0.002	0.006
Rib (experiment)	0.011	0.126	0.003	0.026	0.385	1.251	0.011	0.034
Rib (simulation)	0.024	0.142	0.011	0.028	0.446	1.411	0.004	0.014
Seed (experiment)	0.074	0.020	0.010	0.009	1.148	0.568	0.027	0.017
Seed (simulation)	0.128	0.057	0.066	0.021	0.940	0.693	0.010	0.005

Supplementary Table 9. List of parameters obtained by fitting the constitutive model to experimental and simulation data representing fabric made from the acrylic yarn.

SUPPLEMENTARY NOTE 8. Composite elasticity from reduced-symmetry model (RS model)

To connect between the micromechanics of the yarn and the fabric's macroscopic response, we developed a reduced-symmetry (RS) model of stitch mechanics. This model starts with the full 3D elastica model and determines the change in bending energy due to deflecting the shape of individual yarn segments from their original shape, as they sit in an un-stretched sample of fabric. We distill this shape-response to stretching into a dependence on the spatial symmetry of yarn joining neighboring entangled regions. To this end, we approximate the yarn segment shapes as 2D curves, given by their projections onto either the xz-plane or the yz-plane (as shown in Supplementary Fig. 10). We can approximate the shape of these 3D curves as the image of 2D parametric curves $\mathbf{r}(u) = r_{\parallel}(u)\hat{\mathbf{e}}_{\parallel} + r_{\perp}\hat{\mathbf{e}}_{\perp}$ where $-1/2 \leq u \leq 1/2$, $\hat{\mathbf{e}}_{\parallel}$ is a unit vector in the xy-plane and $\hat{\mathbf{e}}_{\perp} = \hat{\mathbf{z}}$ lies along the fabric's thickness. Translating the coordinate system such that the endpoints of a segment are at antipodal values $\mathbf{r}(\pm 1/2) = \pm \mathbf{r}_0 = \frac{\lambda}{2}(\cos\phi\, \hat{\mathbf{e}}_{\parallel} + \sin\phi\, \hat{\mathbf{e}}_{\perp})$, where λ is the separation of the endpoints. We recognize that there are distinct curves with even symmetry $r_{\text{even}}(-u) = r_{\text{even}}(u)$ (and $\phi = 0$), and distinct curves with odd symmetry $r_{\text{odd}}(-u) = -r_{\text{odd}}(u)$ (and general $\phi \neq 0$). This follows from the mapping of knit stitches to purl stitches in 3D space induced by the action of the mirror operation $M = (\mathbb{1} - 2\hat{\mathbf{e}}_{\perp} \otimes \hat{\mathbf{e}}_{\perp})$ on the centerline of the yarn.

The geometry of the entangled regions at the endpoints of the yarn segment constrains the shape of the curve at its endpoints by the requirement that the curve must clasp around another curve in the entangled region. The end of the yarn is forced to deflect out of the plane, following a given tangent vector $\hat{\mathbf{v}}$ adding an additional set of bound-

	Y_x (N/mm)	Y_y (N/mm)	ν_{yx}	ν_{xy}
Stockinette (experiment)	0.165 ± 0.011	0.753 ± 0.034	0.444 ± 0.005	0.430 ± 0.015
Stockinette (simulation)	0.182	0.684	0.453	0.202
Garter (experiment)	0.223 ± 0.021	0.056 ± 0.015	0.481 ± 0.004	0.150 ± 0.003
Garter (simulation)	0.200	0.030	0.504	0.407
Rib (experiment)	0.010 ± 0.006	0.119 ± 0.012	0.208 ± 0.001	0.294 ± 0.006
Rib (simulation)	0.022	0.129	0.200	0.461
Seed (experiment)	0.070 ± 0.007	0.019 ± 0.001	0.471 ± 0.004	0.138 ± 0.002
Seed (simulation)	0.103	0.046	0.373	0.515

Supplementary Table 10. List of parameters obtained by fitting the Young's moduli and Poisson ratios to experimental and simulation data representing fabric made from the acrylic yarn.

ary conditions to the parametric curve $\mathbf{r}(u)$. For even curves, this boundary condition is $[\partial_u \mathbf{r}_{\text{even}}/|\partial_u \mathbf{r}_{\text{even}}|]_{\pm 1/2} = \hat{v}_{\|}\hat{\mathbf{e}}_{\|} \pm \hat{v}_{\perp}\hat{\mathbf{e}}_{\perp}$ and for odd curves, it is $[\partial_u \mathbf{r}_{\text{odd}}/|\partial_u \mathbf{r}_{\text{odd}}|]_{\pm 1/2} = \hat{\mathbf{v}}$.

To simplify calculations, we will express the curve $\mathbf{r}(u)$ as a dimensionless deflection $\zeta(u)$ transverse to the end-to-end orientation $\boldsymbol{\lambda}(\phi) = \lambda(\cos\phi\,\hat{\mathbf{e}}_{\|} + \sin\phi\,\hat{\mathbf{e}}_{\perp})$ via

$$\mathbf{r}(u) = u\boldsymbol{\lambda}(\phi) + \lambda\zeta(u)\hat{\mathbf{r}}_{0,\perp}(\phi) \qquad \text{(Supplementary Equation 17)}$$

where $\hat{\boldsymbol{\zeta}} = -\sin\phi\,\hat{\mathbf{e}}_{\|} + \cos\phi\,\hat{\mathbf{e}}_{\perp}$ is the direction transverse to the end-to-end orientation. In this representation, the deflection function $\zeta(u)$ obeys the boundary conditions $\zeta(\pm 1/2) = 0$. We additionally take the small-deflection approximation so that the tangent vector at each point is given by $\hat{\mathbf{t}} \approx \hat{\boldsymbol{\lambda}} + \hat{\boldsymbol{\zeta}}\partial_u\zeta + \mathcal{O}(\zeta^2)$. Therefore, for even connecting yarn segments, the slope of the deflection function at the ends is given by $\partial_u\zeta_{\text{even}}(\pm 1/2) \approx \pm\hat{\mathbf{v}} \cdot \hat{\boldsymbol{\zeta}} = \pm\hat{v}_{\zeta}$. For odd connecting yarn segments, the slope is given by $\partial_u\zeta_{\text{odd}}(\pm 1/2) \approx \hat{\mathbf{v}} \cdot \hat{\boldsymbol{\zeta}} = \hat{v}_{\zeta}$.

In the small-deflection approximation, the elastica energy is given by

$$E[\zeta, \lambda, T] \approx \frac{\lambda}{2}\int_{-1/2}^{1/2} \mathrm{d}u\, \left[\frac{B}{\lambda^2}(\partial_u^2\zeta)^2 + T(\partial_u\zeta)^2\right] + T(\lambda - L) \qquad \text{(Supplementary Equation 18)}$$

where T is a Lagrange multiplier, a tension that constrains the length of the curve to L. It is useful to rewrite the energy as

$$E[\zeta, \lambda, q] \approx \frac{B}{2\lambda}\int_{-1/2}^{1/2} \mathrm{d}u\, \left[(\partial_u^2\zeta)^2 + 4q^2(\partial_u\zeta)^2 - 8q^2\left(\frac{L}{\lambda} - 1\right)\right]$$

	C^0_{xxxx} (N/mm)	C^0_{yyyy} (N/mm)	C^0_{xxyy} (N/mm)	C^0_{xxyy} (N/mm)	α_{xx}	α_{yy}	β_{xx} (N/mm)	β_{yy} (N/mm)
Stockinette (experiment)	0.147	0.659	0.061	0.277	1.388	2.440	0.007	0.064
Stockinette (simulation)	0.354	0.637	0.127	0.280	1.250	2.102	0.006	0.012
Garter (experiment)	0.057	0.031	0.010	0.017	1.225	0.700	0.020	0.021
Garter (simulation)	0.208	0.052	0.044	0.024	1.093	0.719	0.024	0.016
Rib (experiment)	0.003	0.049	0.001	0.010	0.388	1.439	0.003	0.017
Rib (simulation)	0.009	0.028	0.004	0.006	0.393	1.243	0.002	0.007
Seed (experiment)	0.038	0.044	0.006	0.019	1.351	0.951	0.010	0.022
Seed (simulation)	0.114	0.192	0.019	0.069	1.102	1.212	0.017	0.008

Supplementary Table 11. List of parameters obtained by fitting the constitutive model to experimental and simulation data representing fabric made from the cotton yarn.

(Supplementary Equation 19)

where $q^2 \equiv T\lambda^2/(4B)$ is a dimensionless form of the Lagrange multiplier. The equilibrium deflection $\zeta(u)$ extremizes this energy function so that $\delta E/\delta\zeta = 0$ and therefore solves the differential equation $\partial_u^4\zeta - 4q^2\partial_u^2\zeta = 0$. Even solutions have the form $\zeta_{\text{even}} = a + b\cosh 2qu$ and odd solutions have the form $\zeta_{\text{odd}} = cu + d\sinh 2qu$, where the constants a, b, c, and d are determined by the boundary conditions on ζ_{even} and ζ_{odd}. Inserting these solutions back into the energy functional, the total energy for even connecting yarn segments E_{even} is given by

$$E_{\text{even}}(\hat{v}_\zeta, \lambda, q) \approx \frac{2B}{\lambda}\left[\hat{v}_\zeta^2 q \coth q - 2q^2\left(\frac{L}{\lambda} - 1\right)\right] \quad \text{(Supplementary Equation 20)}$$

and the total energy for odd connecting yarn segments E_{odd} is given by

$$E_{\text{odd}}(\hat{v}_\zeta, \lambda, q) \approx \frac{2B}{\lambda}\left[\hat{v}_\zeta^2 \frac{q^2 \sinh q}{q\cosh q - \sinh q} - 2q^2\left(\frac{L}{\lambda} - 1\right)\right]. \quad \text{(Supplementary Equation 21)}$$

In order for the length constraint to be enforced, the dimensionless Lagrange multiplier q is chosen to solve the equation $\partial E/\partial q = 0$. However, to solve for q, we require the solution to transcendental equations for both even and odd connecting yarn segments. To avoid this, we find it is sufficient to Taylor expand each energy function to quartic order in q, yielding

$$E_{\text{even}}(\hat{v}_\zeta, \lambda, q) \approx \frac{2B}{\lambda}\left[\hat{v}_\zeta^2\left(1 + \frac{q^2}{3} - \frac{q^4}{45} + \mathcal{O}(q^6)\right) - 2q^2\left(\frac{L}{\lambda} - 1\right)\right]$$

	Y_x (N/mm)	Y_y (N/mm)	ν_{yx}	ν_{xy}
Stockinette (experiment)	0.122 ± 0.018	0.545 ± 0.038	0.420 ± 0.003	0.412 ± 0.020
Stockinette (simulation)	0.298	0.536	0.441	0.359
Garter (experiment)	0.051 ± 0.006	0.028 ± 0.002	0.561 ± 0.006	0.177 ± 0.002
Garter (simulation)	0.188	0.047	0.459	0.210
Rib (experiment)	0.003 ± 0.005	0.046 ± 0.006	0.205 ± 0.003	0.289 ± 0.006
Rib (simulation)	0.008	0.026	0.195	0.439
Seed (experiment)	0.035 ± 0.005	0.041 ± 0.003	0.435 ± 0.006	0.165 ± 0.008
Seed (simulation)	0.107	0.180	0.359	0.168

Supplementary Table 12. List of parameters obtained by fitting Young's moduli and Poisson ratios to experimental and simulation data representing fabric made from the cotton yarn.

(Supplementary Equation 22)

and

$$E_{\text{odd}}(\hat{v}_\zeta, \lambda, q) \approx \frac{2B}{\lambda}\left[\hat{v}_\zeta^2\left(3 + \frac{q^2}{5} - \frac{q^4}{175} + \mathcal{O}(q^6)\right) - 2q^2\left(\frac{L}{\lambda} - 1\right)\right]$$

(Supplementary Equation 23)

which yield approximate polynomial equations for the constraining tension q. Using the solution for q, we find effective elastica energies

$$E_{\text{even}}(\hat{v}_\zeta, \lambda) \approx \frac{2B}{\lambda}\left[\frac{9}{4}\hat{v}_\zeta^2 + \frac{45}{\hat{v}_\zeta^2}\left(\frac{L}{\lambda} - 1\right)\left(\frac{L}{\lambda} - 1 - \frac{\hat{v}_\zeta^2}{3}\right)\right] \quad \text{(Supplementary Equation 24)}$$

and

$$E_{\text{odd}}(\hat{v}_\zeta, \lambda) \approx \frac{2B}{\lambda}\left[\frac{19}{4}\hat{v}_\zeta^2 + \frac{175}{\hat{v}_\zeta^2}\left(\frac{L}{\lambda} - 1\right)\left(\frac{L}{\lambda} - 1 - \frac{\hat{v}_\zeta^2}{5}\right)\right] \quad \text{(Supplementary Equation 25)}$$

which include the lowest-order correction to the bending energy arising from the enforced length constraint. The first term of the energy arises from an overall penalty from curvature, so that at fixed endpoint orientation $\hat{\mathbf{v}}$, the internal stress of the curve pushes its endpoint separation λ to higher values. This stress is countered by the second term, representing the cost of concentrating curvature to the endpoints of the curve when the endpoint separation

	C^0_{xxxx} (N/mm)	C^0_{yyyy} (N/mm)	C^0_{xxyy} (N/mm)	C^0_{yyxx} (N/mm)	α_{xx}	α_{yy}	β_{xx} (N/mm)	β_{yy} (N/mm)
Lace-Weight Acrylic								
Stockinette	0.119	1.454	0.130	0.678	0.655	1.870	0.078	0.275
Garter	0.066	0.154	0.034	0.075	0.657	1.380	0.050	0.056
Rib	0.012	0.261	0.009	0.074	0.342	1.573	0.018	0.083
Seed	0.037	0.189	0.023	0.103	0.589	1.451	0.031	0.037
Lace-Weight Blue Mohair								
Stockinette	0.168	0.410	0.134	0.174	0.670	1.644	0.050	0.125
Garter	0.146	0.069	0.075	0.048	0.847	1.020	0.028	0.034
Rib	0.026	0.126	0.020	0.037	0.327	1.323	0.014	0.039
Seed	0.116	0.130	0.060	0.086	0.803	0.986	0.020	0.028
Lace-Weight Cashmere								
Stockinette	0.044	0.309	0.040	0.129	0.622	1.575	0.035	0.098
Garter	0.034	0.060	0.019	0.033	0.619	1.054	0.028	0.028
Rib	0.007	0.090	0.005	0.026	0.304	1.202	0.012	0.037
Seed	0.030	0.038	0.013	0.025	0.676	0.780	0.022	0.025
Lace-Weight Alpaca Mohair								
Stockinette	0.099	0.399	0.870	0.181	0.633	1.721	0.044	0.116
Garter	0.092	0.064	0.044	0.038	0.769	1.032	0.034	0.0311
Rib	0.021	0.152	0.018	0.051	0.389	1.476	0.012	0.051
Seed	0.104	0.076	0.048	0.052	0.817	0.869	0.018	0.026
Lace-Weight Bamboo								
Stockinette	0.015	0.432	0.016	0.186	0.605	2.095	0.012	0.103
Garter	0.023	0.068	0.015	0.037	0.638	1.542	0.018	0.022
Rib	0.005	0.100	0.004	0.030	0.323	1.787	0.008	0.028
Seed	0.019	0.043	0.010	0.025	0.669	1.019	0.014	0.021

Supplementary Table 13. List of parameters obtained by fitting the constitutive model to experimental data from samples made from lace weight yarn.

λ approaches the total length L of the curve. Therefore, there is an endpoint separation λ^* that minimizes the elastica energy so $\partial E/\partial\lambda|_{\lambda^*} = 0$ at fixed endpoint orientation $\hat{\mathbf{v}}$. For even connecting yarn segments, $\lambda^*_{\text{even}}/L \approx 1 - \hat{v}_\zeta^2/6 + \mathcal{O}(\hat{v}_\zeta^4)$, and for odd connecting yarn segments, $\lambda^*_{\text{odd}}/L \approx 1 - \hat{v}_\zeta^2/10 + \mathcal{O}(\hat{v}_\zeta^4)$. We will next assume that under low applied stress, these segments have separation length λ that are almost the energy-minimizing length λ^*. Expanding the elastica energy to second order in $(\lambda - \lambda^*)/\lambda^*$, the even connecting yarn segment energy is approximately

$$E_{\text{even}}(\hat{v}_\zeta, \lambda) \approx \frac{2B}{\lambda}\left[\hat{v}_\zeta^2 + \frac{45}{\hat{v}_\zeta^2}\left(\frac{\lambda - \lambda^*_{\text{even}}}{\lambda^*_{\text{even}}}\right)^2\right] \qquad \text{(Supplementary Equation 26)}$$

	Y_x (N/mm)	Y_y (N/mm)	ν_{yx}	ν_{xy}
Lace-Weight Acrylic				
Stockinette	0.058	0.714	0.466	1.092
Garter	0.049	0.115	0.488	0.518
Rib	0.010	0.208	0.284	0.723
Seed	0.025	0.126	0.545	0.610
Lace-Weight Blue Mohair				
Stockinette	0.111	0.271	0.425	0.800
Garter	0.095	0.100	0.679	0.515
Rib	0.020	0.098	0.291	0.781
Seed	0.077	0.086	0.661	0.514
Lace-Weight Cashmere				
Stockinette	0.027	0.192	0.417	0.912
Garter	0.024	0.041	0.552	0.552
Rib	0.006	0.072	0.286	0.695
Seed	0.021	0.027	0.652	0.445
Lace-Weight Alpaca Mohair				
Stockinette	0.059	0.240	0.454	0.879
GarterGarter	0.065	0.046	0.596	0.482
Rib	0.015	0.109	0.337	0.841
Seed	0.071	0.052	0.683	0.461
Lace-Weight Bamboo				
Stockinette	0.008	0.240	0.430	1.038
Garter	0.015	0.043	0.549	0.653
Rib	0.004	0.074	0.301	0.834
Seed	0.013	0.029	0.598	0.537

Supplementary Table 14. List of parameters obtained by fitting the Young's moduli and Poisson ratios to experimental data from samples made from lace weight yarn.

and the odd connecting yarn segment energy is approximately

$$E_{\text{odd}}(\hat{v}_\zeta, \lambda) \approx \frac{6B}{\lambda}\left[\hat{v}_\zeta^2 + \frac{175}{3\hat{v}_\zeta^2}\left(\frac{\lambda - \lambda^*_{\text{odd}}}{\lambda^*_{\text{odd}}}\right)^2\right] \qquad \text{(Supplementary Equation 27)}$$

where we have kept only leading-order terms in $\hat{v}_\zeta$. Note that the cost of deforming each segment diverges when the endpoint orientations approach the orientation of the endpoint separation vector, i.e. $\hat{v}_\zeta \to 0$. In this limit, the energy-minimizing length approaches the total length of the curve, $\lambda^* \to L$, and due to the length constraint, the cost of stretching the curve beyond its total length should diverge. Finally, the bending energy for odd connecting yarn segments is generally larger than the bending energy for even connecting yarn segments, assuming each curve has identical values of length L, endpoint separation λ, and endpoint

orientation $\hat{v}_\zeta$. This is reasonable since odd connecting yarn segments have two arches, each with a fraction of the radius of curvature of the single arch of an even connecting yarn segment.

Next, we determine the rigidity $Y \equiv (\partial^2 E/\partial\lambda_\parallel^2)_{\hat{\mathbf{v}}}$ for extensile deformations of each curve along the fabric plane, where $\lambda_\parallel = \boldsymbol{\lambda}\cdot\hat{\mathbf{e}}_\parallel = \lambda\cos\phi$ is the x-axis projection of the endpoint separation. As shown in Supplementary Fig. 10, the endpoint orientation $\hat{\mathbf{v}}$ remains effectively fixed under such deformations. For even connecting yarn segments, the planar projection of the endpoint separation $\lambda_\parallel$ is identical to the full endpoint separation λ, since $\phi = 0$, so $Y_{\mathrm{even}} \equiv (\partial^2 E_{\mathrm{even}}/\partial\lambda^2)_{\hat{\mathbf{v}}}$. Therefore, the extensional rigidity for even connecting yarn segments is approximately

$$Y_{\mathrm{even}} \approx \frac{180B}{\hat{v}_\zeta^2 L\lambda^2} = \frac{180B}{\hat{v}_\zeta^2\lambda^3(1-\delta_{\mathrm{even}})} \qquad \text{(Supplementary Equation 28)}$$

where the divergence as $\hat{v}_\zeta \to 0$ is due to infinite energy cost for stretching the curve beyond its constrained length. Here, $\delta_{\mathrm{even}} = 1-(L/\lambda)$ is a geometric factor. In general, odd connecting yarn segments align along an angle $\phi \neq 0$ and deformations of the x-axis projection of the endpoint separation $\lambda_\parallel$ can be achieved by changes in both the endpoint separation λ and the angle ϕ. It is evident that deformations involving changes in endpoint separation λ are even more rigid than those for even connecting yarn segments. Therefore, odd connecting yarn segments undergo extensile deformations by rotating into the fabric plane (as shown in Supplementary Fig. 10b), i.e. they change $\lambda_\parallel$ by changing the angle ϕ at fixed λ so $Y_{\mathrm{odd}} \equiv (\partial^2 E_{\mathrm{odd}}/\partial\lambda_\parallel^2)_{\hat{\mathbf{v}},\lambda}$. Therefore, the extensional rigidity for odd connecting yarn segments is approximately

$$Y_{\mathrm{odd}} \approx \frac{12B}{L\lambda_\perp^2}(1+\hat{v}_\zeta\cot\phi) = \frac{12B}{\lambda^3(1-\delta_{\mathrm{odd}})}(1+\hat{v}_\zeta\cot\phi) \quad \text{(Supplementary Equation 29)}$$

where $\lambda_\perp = \boldsymbol{\lambda}\cdot\hat{\mathbf{e}}_\perp = \lambda\sin\phi$ is the out-of-plane projection of the endpoint separation λ, and $\delta_{\mathrm{odd}} = 1-(L\sin\phi/\lambda)$. Note that this energy diverges as $\phi \to 0$, for which the rotational freedom of the odd connecting yarn segments saturates and the curve must increase λ in order to undergo extensile deformations, much like even connecting yarn segments. However, for garter, rib, and seed stitches, values of ϕ for the un-deformed stitch are closer to 45°, so that $\cot\phi = 1$. In this case, we find that the ratio of even connecting yarn segments rigidity to odd connecting yarn segments rigidity can be significantly greater than one, with $Y_{\mathrm{even}}/Y_{\mathrm{odd}} \sim \mathcal{O}(10)$ being typical.

8.1. *Effective stitch rigidities*

The RS model provides estimates for the linear stiffnesses of different yarn connecting segments, based on the “rule of mixing” from the theory of fiber composites. To estimate the effective linear stiffness of the entire stitch, we treat each connecting segment as a spring element, either in series or in parallel with other springs comprising the stitch, as shown in Supplementary Fig. 10. For stretching in the x-direction, we consider the effective stiffness of the connecting yarn segments that are oriented in the x-direction; likewise for stretching in the y-direction. Like-stitch neighbors (**K**-**K** or **P**-**P**) have a pair of spring elements, each with stiffness Y_{even}, that add in series in the x-direction, giving an effective stiffness of $Y_{\mathrm{eff}} = Y_{\mathrm{even}}/2$; in the y-direction, two sets of in-series pairs are in parallel, giving

an effective stiffness of $Y_{\text{eff}} = Y_{\text{even}}$. Unlike-stitch neighbors (**K**-**P**) have different spring elements in the x-direction and y-direction. In the x-direction, there is one even connecting yarn segment in series with an odd connecting yarn segment, leading to a effective stiffness of $Y_{\text{eff}} = (Y_{\text{even}}^{-1} + Y_{\text{odd}}^{-1})^{-1} \approx Y_{\text{odd}}$, since $Y_{\text{odd}}/Y_{\text{even}} \ll 1$. In the y-direction, the springs add similarly to the like-stitch case, giving an effective stiffness of $Y_{\text{eff}} = Y_{\text{odd}}$.

	λ (mm)	δ	$\hat{v}_\zeta$	ϕ
Stockinette (x; even)	2.187	-0.884	1.000	-
Stockinette (y; even)	5.289	-0.490	0.686	-
Garter (x; even)	2.120	-0.583	1.000	-
Garter (y; odd)	3.499	0.257	0.686	0.749
Rib (x; odd)	3.093	-0.156	1.000	1.181
Rib (y; even)	4.846	-0.334	0.999	-
Seed (x; odd)	3.053	0.462	0.073	0.450
Seed (y; odd)	3.776	-0.020	0.720	0.821

Supplementary Table 15. List of geometric parameters for use in RS model calculations, obtained from simulations of acrylic yarn.

8.2. The high-tension limit

As the endpoints $\pm\mathbf{r}_0$ are brought further apart, the distribution of curvature in the elastica shifts and becomes increasingly concentrated at the endpoints, as the central segment straightens out. Since the curve must maintain a fixed length, the force required to move these points apart increases until the distance between the points is equal to the total length of the curve. In this pathological limit, the radius of curvature at the endpoints approaches zero and the bending energy diverges; the tension T required to maintain the fixed-length constraint likewise diverges. The nature of this strain-stiffening response can therefore be obtained in the asymptotic $T \to \infty$ limit of the elastica model. This is equivalent to taking the large-q limit in the evaluation of the energies Supplementary Equation 20 and Supplementary Equation 21. In this limit, the integrands of the energy functionals for both even and odd connecting yarn segments have the same q-dependence. Minimizing each energy with respect to q, we find an identical asymptotic form to the energy,

$$E_{\text{high-tension}}(\hat{v}_\zeta, \lambda) \simeq \frac{B}{4L}\frac{\hat{v}_\zeta^4}{1-\frac{\lambda}{L}}. \qquad \text{(Supplementary Equation 30)}$$

	λ (mm)	δ	$\hat{v}_\zeta$	ϕ
Stockinette (x; even)	2.442	-0.421	0.988	-
Stockinette (y; even)	3.559	-0.513	0.783	-
Garter (x; even)	3.488	-0.493	1.000	-
Garter (y; odd)	4.895	0.202	0.696	0.770
Rib (x; odd)	2.834	-0.565	1.000	1.355
Rib (y; even)	5.744	-0.338	1.000	-
Seed (x; odd)	3.344	0.432	0.100	0.474
Seed (y; odd)	4.181	-0.102	0.816	0.864

Supplementary Table 16. List of geometric parameters for use in RS model calculations, obtained from simulations of cotton yarn.

Thus, we recover both the expected strain-stiffening response $f(\lambda) \sim (1-\lambda/L)^{-2}$ and the observed universal strain-stiffening behavior, independent of stitch symmetry.

SUPPLEMENTARY NOTE 9. Applying the constitutive model to simulate uniaxial stretching in fabrics of finite size

To simulate the deformed shape of sample of fabric of finite extent using our constitutive model (Supplementary Equation 14), we turn to continuum elasticity theory.

There is considerable prior work on numerical homogenization of yarn level simulations that use micromechanical simulations to predict the bulk level elastic response that is then implemented in FEA [25–27].We emphasize here that our FEA calculation is a proof of concept demonstration that the nonlinear constitutive model captures realistic fabric-level deformations. Rather than the micromechanical model being tied directly into the FEA calculation, we use the elastica model to derive the nonlinear form of the constitutive model, based on stitch microstructure. Here, we use constitutive model coefficients that are found from fitting experimental (rather than simulation) data.

We will denote points inside the undeformed fabric as $\mathbf{r} = (x, y)$, where $x \in [-W_0/2, W_0/2]$ and $y \in [-L_0/2, L_0/2]$. The dimensions of the undeformed garter fabric made of acrylic yarn, measured prior to the stretching experiment, are $W_0 = 127\,\mathrm{mm}$ and $L_0 = 40\,\mathrm{mm}$. Under applied uniaxial displacement of the boundaries, fabric points $\mathbf{r}$ are displaced by a vector field $\mathbf{u}(\mathbf{r})$ to new points $\mathbf{R}(\mathbf{r}) = \mathbf{r} + \mathbf{u}(\mathbf{r})$, resulting in a linearized strain tensor

$$\varepsilon_{ij}(\mathbf{r}) = \frac{1}{2}\left(\partial_i u_j + \partial_j u_i\right) . \qquad \text{(Supplementary Equation 31)}$$

	Y_x (N/mm)	Y_y (N/mm)	ν_{yx}	ν_{xy}
Stockinette (experiment)	0.165 ± 0.011	0.753 ± 0.034	0.444 ± 0.005	0.430 ± 0.015
Stockinette (simulation)	0.182	0.684	0.453	0.202
Stockinette (RS model)	0.210	0.528	-	-
Garter (experiment)	0.223 ± 0.021	0.056 ± 0.015	0.481 ± 0.004	0.150 ± 0.003
Garter (simulation)	0.200	0.030	0.504	0.407
Garter (RS model)	0.275	0.106	-	-
Rib (experiment)	0.010 ± 0.006	0.119 ± 0.012	0.208 ± 0.001	0.294 ± 0.006
Rib (simulation)	0.022	0.129	0.200	0.461
Rib (RS model)	0.024	0.109	-	-
Seed (experiment)	0.070 ± 0.007	0.019 ± 0.001	0.471 ± 0.004	0.138 ± 0.002
Seed (simulation)	0.103	0.046	0.373	0.515
Seed (RS model)	0.077	0.029	-	-

Supplementary Table 17. List of parameters obtained by fitting the Young's moduli and Poisson ratios to experimental and simulation data representing fabric made from the acrylic yarn. Also included: estimates of stitch rigidity from the reduced-symmetry (RS) elastica model using geometric parameters obtained from simulations of relaxed stitches.

This strain corresponds to an internal stress field $\sigma_{ij}(\mathbf{r})$ via our constitutive model (Supplementary Equation 14). The points $\mathbf{r}$ and $\mathbf{R}$ lie on two-dimensional triangular meshes with the same topology (no re-meshing is performed during the calculation; see Supplementary Fig. 12). The final shape adopted by the fabric under set displacements of the y-boundary, $\mathbf{u}(x, \pm L_0/2) = \pm U\hat{\mathbf{y}}$, is determined by solving the continuum elasticity equilibrium equations,

$$\partial_j \sigma_{ij}(\mathbf{r}) = 0\,, \qquad \text{(Supplementary Equation 32)}$$

with boundary conditions $\sigma_{xj}(\pm W_0/2, y) = 0$ on the x-boundary.

To solve this boundary value problem, we turn to Finite Element Analysis (FEA), as implemented by FEniCS, an open-source finite element solver (see `https://fenicsproject.org/`). Rather than directly solving the stress balance partial differential equation, the

	Y_x (N/mm)	Y_y (N/mm)	ν_{yx}	ν_{xy}
Stockinette (experiment)	0.122 ± 0.018	0.545 ± 0.038	0.420 ± 0.003	0.412 ± 0.020
Stockinette (simulation)	0.298	0.536	0.441	0.359
Stockinette (RS model)	0.312	0.602	-	-
Garter (experiment)	0.051 ± 0.006	0.028 ± 0.002	0.561 ± 0.006	0.177 ± 0.002
Garter (simulation)	0.188	0.047	0.459	0.210
Garter (RS model)	0.099	0.052	-	-
Rib (experiment)	0.003 ± 0.005	0.046 ± 0.006	0.205 ± 0.003	0.289 ± 0.006
Rib (simulation)	0.008	0.026	0.195	0.439
Rib (RS model)	0.029	0.099	-	-
Seed (experiment)	0.035 ± 0.005	0.041 ± 0.003	0.435 ± 0.006	0.165 ± 0.008
Seed (simulation)	0.107	0.180	0.359	0.168
Seed (RS model)	0.079	0.033	-	-

Supplementary Table 18. List of parameters obtained by fitting Young's moduli and Poisson ratios to experimental and simulation data representing fabric made from the cotton yarn. Also included: estimates of stitch rigidity from the reduced-symmetry (RS) elastica model using geometric parameters obtained from simulations of relaxed stitches.

problem is cast in its "weak form," derived from the energy functional

$$E[\varepsilon] = \int_{-W_0/2}^{W_0/2} \int_{-L_0/2}^{L_0/2} \mathrm{d}x\,\mathrm{d}y \left\{ \frac{1}{2} C^0_{ijkl} \varepsilon_{ij} \varepsilon_{kl} + \frac{\beta_{xx} \alpha_{xx}^2 \varepsilon_{xx}^3}{1 - \alpha_{xx} \varepsilon_{xx}} + \frac{\beta_{yy} \alpha_{yy}^2 \varepsilon_{yy}^3}{1 - \alpha_{yy} \varepsilon_{yy}} \right\} .$$

(Supplementary Equation 33)

Note that this energy functional requires the symmetry $C^0_{xxyy} = C^0_{yyxx}$, whereas our fits show significant asymmetry between these components. To continue using this form of the energy functional, we use the average of the measured values of C^0_{xxyy} and C^0_{yyxx}. Following the standard FEA procedure, we create a meshed representation of the undeformed fabric. The mesh elements at the top and bottom boundaries of the fabric are displaced by the fixed boundary displacement U. Next, the program calculates the variations in the total

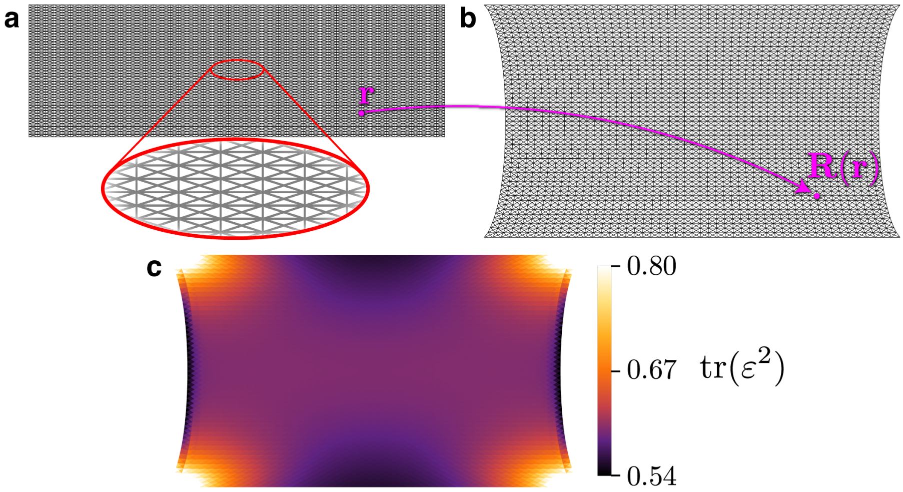

Supplementary Fig. 12. (**a**) Triangular mesh of the undeformed fabric (reference state) used for FEA calculations, with inset showing detailed mesh structure and $\mathbf{r}$ representing an arbitrary point on the mesh. (**b**) Deformed state of the same mesh, where $\mathbf{R}(\mathbf{r})$ is the image of $\mathbf{r}$ under the uniaxial stretch deformation. (**c**) Local deformation, as quantified by $\mathrm{tr}(\varepsilon^2)$.

energy δE with respect to displacements of the mesh vertices δu_k. Finally, the program iteratively searches for the root $\delta E(\mathbf{u}) = 0$. To avoid numerical issues due to the singular form of the nonlinear part of the elastic energy functional, we approximate the nonlinear part by its series expansion, truncating at quartic order (dropping terms $\mathcal{O}(\varepsilon^5)$ and higher). While we did not calculate the elastic constant C_{xyxy} in experiments or simulations, for this demonstration, we chose $C_{xyxy} = 0.01$ N mm^{-1}, which is on a comparable scale as the other elastic constants. Since the majority of the uniaxial deformation involves ε_{xx} and ε_{yy} components of strain, the simulated deformations are relatively insensitive to this one elastic constant.

SUPPLEMENTARY NOTE 10. Therapeutic Glove Prototype

We fabricated the samples of different fabrics by hand using 2.75 mm and 2.0 mm needles. We then measured the stiffness of each test sample using the uniaxial stretching experiment protocol, described in SUPPLEMENTARY NOTE 1. The experiment results are shown in Supplementary Fig. 13, constitutive model fits are given in Supplementary Table 19, and the Young's moduli and Poisson ratios are given in Supplementary Table 20. The yarns used for the glove were chosen because visually they would help the reader discern which stitch patterns were implemented and where. These yarns were fairly similar to the acrylic yarn, which was unavailable in a large enough color variety for this experiment.

We took measurements of the hand, in particular the relative location of joints and other parts of the anatomy. We used those measurements to determine where in the glove we needed rigidity and where we needed flexibility to support natural hand motion for the specific glove wearer. This determined which type of fabric was needed in each region of the glove. We used the stitch gauge – how many stitches comprise five centimeters of fabric, also known as the stitch density – from the test samples to determine of the number of stitches needed in each part of the glove to match the hand dimensions. We targeted 20 - 30

mm Hg for the hand of the specific wearer but it was calculated to be approximately 3.75 kPa or 28 mm Hg. This pressure was calculated by measuring the rest, flat position of the circumference of the glove, then measuring the circumference of the glove on the the hand. From these measurements, we calculate the linear strain of the glove as it is being worn. Using Supplementary Fig. 13, we use this strain to find a correlated stress. Multiplying the stress by the width of the wrist support segment (the stockinette region around the wrist) gives a force, which is then divided by the area of the wrist support segment to estimate a pressure. The pressure provided by the theraputic glove prototype is comparable to the pressure that compression stockings are, between 30-40 mm Hg [28]. We have achieved this comparable pressure without the use of elastane. The placement of the other stitch patterns was chosen *ad hoc* to illustrate the anisotropic behavior of the fabric to enable unrestricted motion of the human hand.

To create a seamless pattern which augments the stiffness of the stockinette fabric that supports the radiocarpal and intercarpal joints, we chose to knit the pattern as a single flat piece, starting from the thumb, wrapping the hand from the back to the front, and finally grafting the start and end of the fabric together into a glove. Knitting this horizontally as a flat piece rather than as a tubular knit enabled us to exploit the stiffest direction of stockinette fabric to provide pressure to the wrist. For the illustration in this paper, we chose to highlight the different types of fabric with different-colored yarn. These were knitted together *in situ* using a seamless joining technique known as "intarsia." The pattern shown in Supplementary Fig. 14 can also be knit with a single color of yarn without using intarsia.

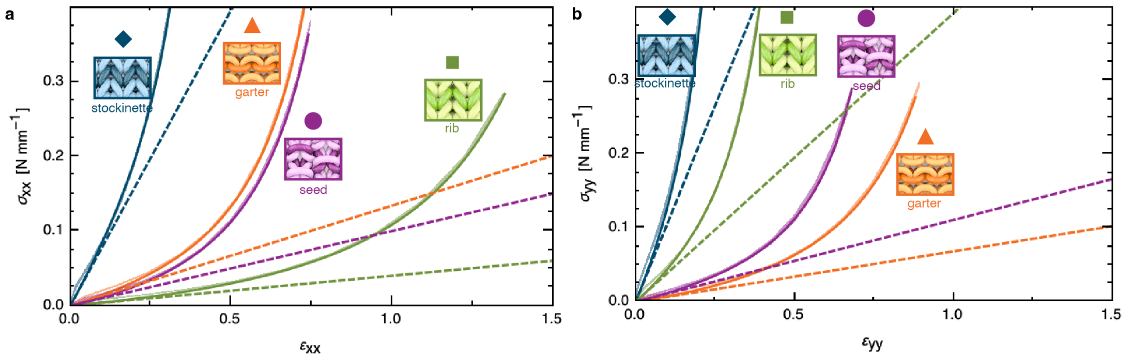

Supplementary Fig. 13. The experimental stress-versus-strain relations for the four fabrics made for the therapeutic glove prototype in the (**a**) x- and (**b**) y-directions. All of the data for each type of fabric is displayed by a different color: stockinette in blue, garter in orange, rib in green, and seed in purple. The experimental data is shown in the translucent regions where the width of the region is one standard deviation of the data. The solid curves are fits to the constitutive relations. Dashed lines depict the linear response at zero stress.

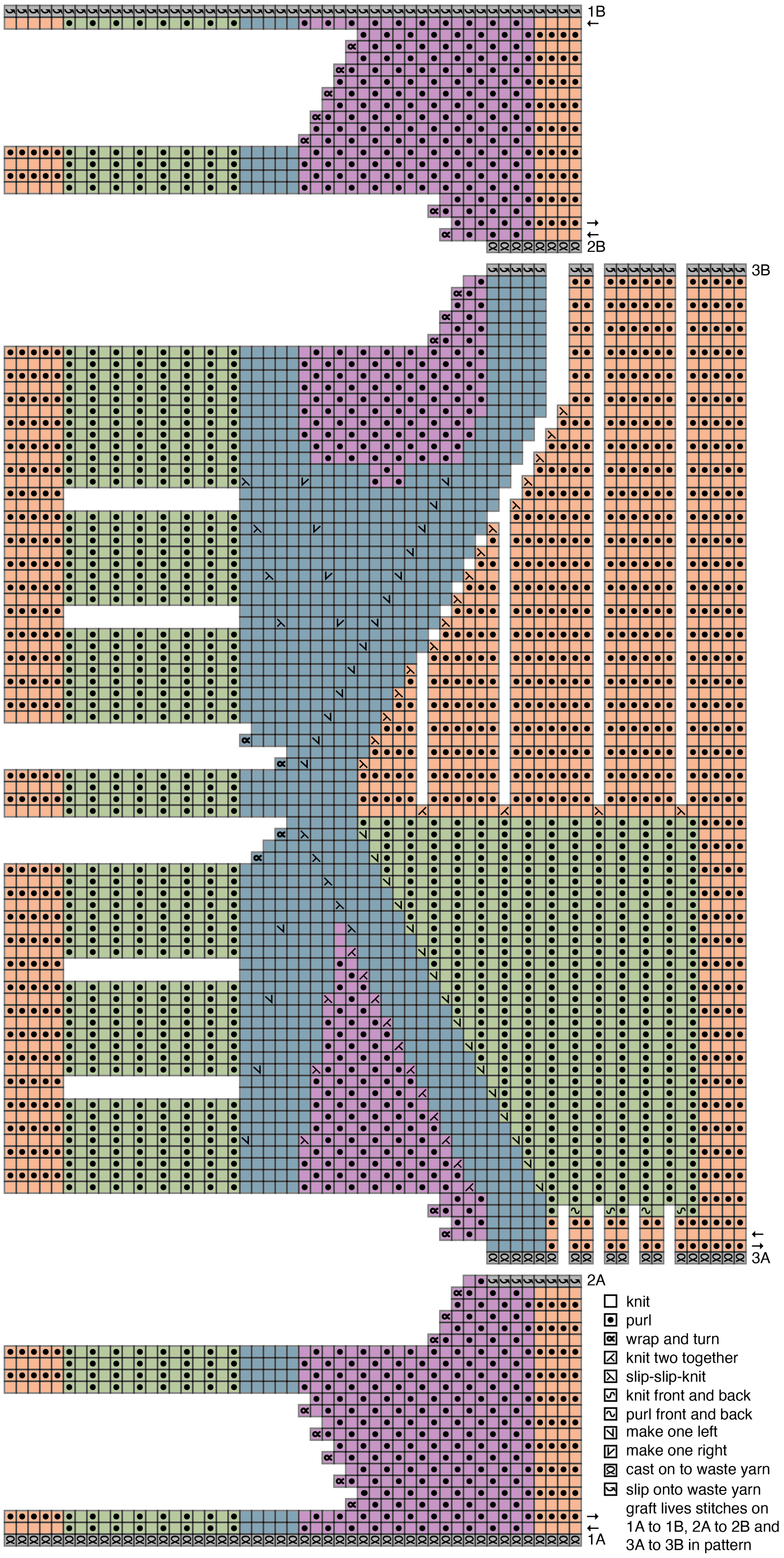

Supplementary Fig. 14. Pattern for the therapeutic glove. Arrows indicate direction of knitting.

	C^0_{xxxx} (N/mm)	C^0_{yyyy} (N/mm)	C^0_{xxyy} (N/mm)	C^0_{yyxx} (N/mm)	α_{xx}	α_{yy}	β_{xx} (N/mm)	β_{yy} (N/mm)
Stockinette	0.210	0.590	0.116	0.267	0.926	1.777	0.045	0.047
	1.225*	1.646*	0.758*	0.936*	1.806*	2.426*	0.051*	0.088*
Garter	0.149	0.076	0.035	0.032	0.928	0.732	0.043	0.039
Rib	0.046	0.452	0.028	0.100	0.483	1.547	0.039	0.059
Seed	0.108	0.120	0.225	0.044	0.889	1.006	0.047	0.028

Supplementary Table 19. List of parameters obtained by fitting the constitutive model to experimental data representing test samples made for the therapeutic glove. Data that is starred was made on 2.00 mm knitting needles (US size 0) and all remaining data was knit on 2.75 mm knitting needles (US size 2).

	Y_x (N/mm)	Y_y (N/mm)	ν_{yx}	ν_{xy}
Stockinette	0.157	0.442	0.452	0.554
	0.793*	1.066*	0.569*	0.619*
Garter	0.134	0.068	0.419	0.236
Rib	0.040	0.392	0.222	0.599
Seed	0.100	0.111	0.362	0.208

Supplementary Table 20. List of parameters obtained by fitting the Young's moduli and Poisson ratios to experimental data representing the test samples of the therapeutic glove. Data that is starred was made on 2.00 mm knitting needles (US size 0) and all remaining data was knit on 2.75 mm knitting needles (US size 2).

SUPPLEMENTARY NOTE 11. Knitting Machine versus Hand Knitting

In general, ensuring uniform tension and uniform stitch size between types of fabrics is challenging for knitting. We were able to uniformly craft and replicate each fabric type with an equivalent number of rows and columns with a knitting machine. The knitting machine is ideal for ensuring uniform tension throughout the sample; however, it comes at the expense of not guaranteeing uniform stitch size between types of fabrics. For the lace weight samples made with the STOLL Industrial knitting machine, stockinette and garter were made with a stitch size setting of 12 while rib and seed were made at size 11. We find that if all four types of fabric are made at size 12, rib and seed are significantly more loose (Supplementary Fig. 15). Hand knitting, in contrast, cannot guarantee uniform tension throughout the sample but results in more uniform stitch size even while altering the pattern of the knit and purl stitches due to the fixed diameter of the knitting needle, as seen in Supplementary Table 2. Despite these differences, we do get consistent behavior of the four different types of fabric both machine made (Fig. 2 and Supplementary Fig. 2) and hand made (Supplementary Fig. 13). Supplementary Table 2 displays the differences in the

yarn per stitch and the yarn diameter between machine-knit and hand-knit samples for the acrylic yarn.

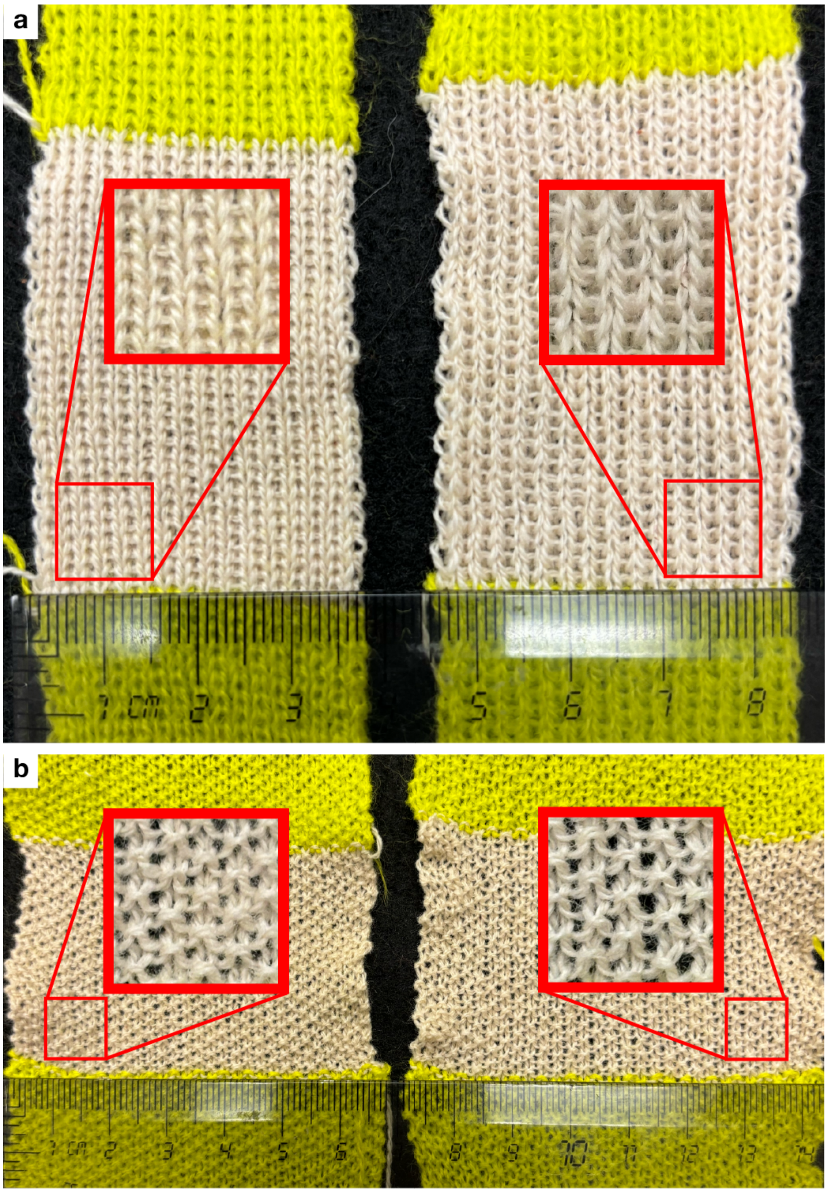

Supplementary Fig. 15. Comparison of (**a**) rib and (**b**) seed fabrics made on the STOLL Industrial knitting machine at different assigned stitch sizes. On the left are fabrics made at size 11 whereas on the right they are made at size 12. The insets display a closeup on a 1 cm by 1 cm portion of the fabrics.

---

[1] Landau, L. D., Lifshitz, E. M., Kosevich, A. M. & Pitaevskii, L. P. *Theory of Elasticity*. Course of theoretical physics (Butterworth-Heinemann, 1986).

[2] Cornelissen, B. & Akkerman, R. Analysis of yarn bending behaviour. *IEEE Transactions on Biomedical Engineering - IEEE TRANS BIOMED ENG* (2009).

[3] Kaldor, J. M., James, D. L. & Marschner, S. Simulating knitted cloth at the yarn level. In *ACM SIGGRAPH 2008 Papers*, SIGGRAPH '08, DOI: 10.1145/1399504.1360664 (Association for Computing Machinery, New York, NY, USA, 2008).

[4] Sperl, G., Sánchez-Banderas, R. M., Li, M., Wojtan, C. & Otaduy, M. A. Estimation of yarn-level simulation models for production fabrics. *ACM Transactions on Graphics* **41**, 1–15, DOI: 10.1145/3528223.3530167 (2022).

[5] Park, J.-W. & Oh, A.-G. Bending Rigidity of Yarns. *Textile Research Journal* **76**, 478–485, DOI: 10.1177/0040517506063915 (2006).

[6] Vassiliadis, S., Kallivretaki, A. & Provatidis, C. Mechanical simulation of the plain weft knitted fabrics. *International Journal of Clothing Science and Technology* **19**, 109–130, DOI: 10.1108/09556220710725711 (2007).

[7] Liu, D. *et al.* On the role of material architecture in the mechanical behavior of knitted textiles. *International Journal of Solids and Structures* **109**, 101–111, DOI: https://doi.org/10.1016/j.ijsolstr.2017.01.011 (2017).

[8] Cirio, G., Lopez-Moreno, J. & Otaduy, M. A. Yarn-level cloth simulation with sliding persistent contacts. *IEEE Transactions on Visualization and Computer Graphics* **23**, 1152–1162, DOI: 10.1109/TVCG.2016.2592908 (2017).

[9] Liu, D., Shakibajahromi, B., Dion, G., Breen, D. & Kontsos, A. A Computational Approach to Model Interfacial Effects on the Mechanical Behavior of Knitted Textiles. *Journal of Applied Mechanics* **85**, DOI: 10.1115/1.4039046 (2018).

[10] Postle, R. & Munden, D. L. 24—ANALYSIS OF THE DRY-RELAXED KNITTED-LOOP CONFIGURATION: PART i: TWO-DIMENSIONAL ANALYSIS. *Journal of the Textile Institute* **58**, 329–351, DOI: 10.1080/00405006708629880 (1967).

[11] Semnani, D., Latifi, M., Hamzeh, S. & Jeddi, A. A new aspect of geometrical and physical principles applicable to the estimation of textile structures: An ideal model for the plain-knitted loop. *Journal of the Textile Institute* **94**, 202–211, DOI: 10.1080/00405000308630609 (2003).

[12] Ramgulam, R. 3 - modelling of knitting. In Au, K. (ed.) *Advances in Knitting Technology*, Woodhead Publishing Series in Textiles, 48–85, DOI: https://doi.org/10.1533/9780857090621.1.48 (Woodhead Publishing, 2011).

[13] Abel, J., Luntz, J. & Brei, D. A two-dimensional analytical model and experimental validation of garter stitch knitted shape memory alloy actuator architecture. *Smart Materials and Structures* **21**, 085011, DOI: 10.1088/0964-1726/21/8/085011 (2012).

[14] Kyosev, Y. K. 6 - the finite element method (FEM) and its application to textile technology. In Veit, D. (ed.) *Simulation in Textile Technology*, Woodhead Publishing Series in Textiles, 172–222e, DOI: https://doi.org/10.1533/9780857097088.172 (Woodhead Publishing, 2012).

[15] Abghary, M. J., Hasani, H. & Nedoushan, R. J. Numerical simulating the tensile behavior of 1×1 rib knitted fabrics using a novel geometrical model. *Fibers and Polymers* **17**, 795–800, DOI: 10.1007/s12221-016-5791-6 (2016).

[16] Htoo, N. N., Soga, A., Wakako, L., Ohta, K. & Kinari, T. 3-dimension simulation for loop structure of Weft Knitted fabric considering mechanical properties of yarn. *Journal of Fiber Science and Technology* **73**, 105–113, DOI: 10.2115/fiberst.2017-0015 (2017).

[17] Sha, S. *et al.* Review on the 3-d simulation for weft knitted fabric. *Journal of Engineered Fibers and Fabrics* **16**, 15589250211012527, DOI: 10.1177/15589250211012527 (2021). https://doi.org/10.1177/15589250211012527.

[18] Eck, M. & Lasser, D. B-spline-bézier representation of geometric spline curves: Quartics and quintics. *Computers & Mathematics with Applications* **23**, 23–39, DOI: 10.1016/0898-1221(92)90066-Q (1992).

[19] DeBenedictis, A. & Atherton, T. J. Shape minimisation problems in liquid crystals. *Liquid Crystals* **43**, 2352–2362, DOI: 10.1080/02678292.2016.1209699 (2016).

[20] Duhovic, M. & Bhattacharyya, D. Simulating the deformation mechanisms of knitted fabric composites. *Composites Part A: Applied Science and Manufacturing* **37**, 1897–1915, DOI: 10.1016/j.compositesa.2005.12.029 (2006).

[21] Postle, R. Structural mechanics of knitted fabrics for apparel and composite materials. *International Journal of Clothing Science and Technology* **14**, 257–268, DOI: 10.1108/09556220210437239 (2002).

[22] Pieranski, P., Strzelecki, L. & Pansu, B. Thin colloidal crystals. *Physical Review Letters* **50**, 900–903, DOI: 10.1103/PhysRevLett.50.900 (1983).

[23] Ru, X., Wang, J. C., Peng, L., Shi, W. & Hu, X. Modeling and deformation simulation of weft knitted fabric at yarn level. *Textile Research Journal* **93**, 2437–2448, DOI: 10.1177/00405175221134934 (2023). _eprint: https://doi.org/10.1177/00405175221134934.

[24] Sokolnikoff, I. Mathematical theory of elasticity, mcgraw-hill book company. *Inc., New York/London* (1956).

[25] Sperl, G., Narain, R. & Wojtan, C. Homogenized yarn-level cloth. *ACM Trans. Graph.* **39**, DOI: 10.1145/3386569.3392412 (2020).

[26] Wadekar, P., Perumal, V., Dion, G., Kontsos, A. & Breen, D. An optimized yarn-level geometric model for Finite Element Analysis of weft-knitted fabrics. *Computer Aided Geometric Design* **80**, 101883, DOI: 10.1016/j.cagd.2020.101883 (2020).

[27] Liu, D., Koric, S. & Kontsos, A. A Multiscale Homogenization Approach for Architectured Knitted Textiles. *Journal of Applied Mechanics* **86**, 111006, DOI: 10.1115/1.4044014 (2019).

[28] Lim, C. S. & Davies, A. H. Graduated compression stockings. *CMAJ* **186**, E391–E398, DOI: 10.1503/cmaj.131281 (2014). https://www.cmaj.ca/content/186/10/E391.full.pdf.
