# Supplementary material for: Programming mechanics in knitted materials, stitch by stitch
Source: Nat Commun. 2024 Mar 23;15:2622. doi: 10.1038/s41467-024-46498-z (PMC10960873; doi:10.1038/s41467-024-46498-z)
Supplement: Supplementary file 3 — Source Data [file 41467_2024_46498_MOESM3_ESM.zip › SourceData/Source Data for Supplementary Information/TableS20.pdf]

	Y_x (N/mm)	Y_y (N/mm)	ν_{yx}	ν_{xy}
Stockinette	0.157	0.442	0.452	0.554
	0.793*	1.066*	0.569*	0.619*
Garter	0.134	0.068	0.419	0.236
Rib	0.040	0.392	0.222	0.599
Seed	0.100	0.111	0.362	0.208
