# Supplementary material for: Programming mechanics in knitted materials, stitch by stitch
Source: Nat Commun. 2024 Mar 23;15:2622. doi: 10.1038/s41467-024-46498-z (PMC10960873; doi:10.1038/s41467-024-46498-z)
Supplement: Supplementary file 3 — Source Data [file 41467_2024_46498_MOESM3_ESM.zip › SourceData/Source Data for Supplementary Information/TableS14.pdf]

	Y_x (N/mm)	Y_y (N/mm)	ν_{yx}	ν_{xy}
Lace-Weight Acrylic				
Stockinette	0.058	0.714	0.466	1.092
Garter	0.049	0.115	0.488	0.518
Rib	0.010	0.208	0.284	0.723
Seed	0.025	0.126	0.545	0.610
Lace-Weight Blue Mohair				
Stockinette	0.111	0.271	0.425	0.800
Garter	0.095	0.100	0.679	0.515
Rib	0.020	0.098	0.291	0.781
Seed	0.077	0.086	0.661	0.514
Lace-Weight Cashmere				
Stockinette	0.027	0.192	0.417	0.912
Garter	0.024	0.041	0.552	0.552
Rib	0.006	0.072	0.286	0.695
Seed	0.021	0.027	0.652	0.445
Lace-Weight Alpaca Mohair				
Stockinette	0.059	0.240	0.454	0.879
GarterGarter	0.065	0.046	0.596	0.482
Rib	0.015	0.109	0.337	0.841
Seed	0.071	0.052	0.683	0.461
Lace-Weight Bamboo				
Stockinette	0.008	0.240	0.430	1.038
Garter	0.015	0.043	0.549	0.653
Rib	0.004	0.074	0.301	0.834
Seed	0.013	0.029	0.598	0.537
