# Supplementary material for: Programming mechanics in knitted materials, stitch by stitch
Source: Nat Commun. 2024 Mar 23;15:2622. doi: 10.1038/s41467-024-46498-z (PMC10960873; doi:10.1038/s41467-024-46498-z)
Supplement: Supplementary file 3 — Source Data [file 41467_2024_46498_MOESM3_ESM.zip › SourceData/Source Data for Supplementary Information/TableS8.pdf]

	E_{total} (J)	$E_{\text{compression}}/E_{\text{total}}$	$E_{\text{bending}}/E_{\text{total}}$
Stockinette (acrylic)	0.249	0.130	0.870
Garter (acrylic)	0.175	0.026	0.974
Rib (acrylic)	0.189	0.391	0.609
Seed (acrylic)	0.288	0.459	0.541
Stockinette (cotton)	0.401	0.233	0.767
Garter (cotton)	0.254	0.216	0.784
Rib (cotton)	0.139	0.044	0.956
Seed (cotton)	0.262	0.172	0.828
