# Supplementary material for: Programming mechanics in knitted materials, stitch by stitch
Source: Nat Commun. 2024 Mar 23;15:2622. doi: 10.1038/s41467-024-46498-z (PMC10960873; doi:10.1038/s41467-024-46498-z)
Supplement: Supplementary file 3 — Source Data [file 41467_2024_46498_MOESM3_ESM.zip › SourceData/Source Data for Supplementary Information/TableS11data/TableS11.pdf]

	C^0_{xxxx} (N/mm)	C^0_{yyyy} (N/mm)	C^0_{xxyy} (N/mm)	C^0_{xxyy} (N/mm)	α_{xx}	α_{yy}	β_{xx} (N/mm)	β_{yy} (N/mm)
Stockinette (experiment)	0.147	0.659	0.061	0.277	1.388	2.440	0.007	0.064
Stockinette (simulation)	0.354	0.637	0.127	0.280	1.250	2.102	0.006	0.012
Garter (experiment)	0.057	0.031	0.010	0.017	1.225	0.700	0.020	0.021
Garter (simulation)	0.208	0.052	0.044	0.024	1.093	0.719	0.024	0.016
Rib (experiment)	0.003	0.049	0.001	0.010	0.388	1.439	0.003	0.017
Rib (simulation)	0.009	0.028	0.004	0.006	0.393	1.243	0.002	0.007
Seed (experiment)	0.038	0.044	0.006	0.019	1.351	0.951	0.010	0.022
Seed (simulation)	0.114	0.192	0.019	0.069	1.102	1.212	0.017	0.008
