# Supplementary material for: Programming mechanics in knitted materials, stitch by stitch
Source: Nat Commun. 2024 Mar 23;15:2622. doi: 10.1038/s41467-024-46498-z (PMC10960873; doi:10.1038/s41467-024-46498-z)
Supplement: Supplementary file 3 — Source Data [file 41467_2024_46498_MOESM3_ESM.zip › SourceData/Source Data for Supplementary Information/TableS11data/README.rtf]

README for Table 11 Raw DataWritten by Sarah E. GonzalezLast Updated February 2 2024Included in this folder is the raw stress strain data used to make the constitutive fits described in Table 11. Experimental data is in the ExperimenalData folder. The files within are organized as follows:stress in x, stress in y, strain in x, strain in y, orientation. The orientation is 0 when the fabric is pulled in the x-direction and 1 if the fabric is pulled in the y-direction.Simulation data is in the SimulationData folder. The files within are organized as follows:stress in x, stress in y, strain in x, strain in y. The orientation is not explicitly included in the data set. Instead, the fabric is pulled int he x-direction when the stress in y=0 and is pulled in the y-direction when the stress in x=0.The uniaxial data is provided in the data for Fig S2, but it can also be derived from this data set. To get the transverse data, plot stress in x versus strain in y and stress in y versus strain in x. Using all the uniaxial data and all the transverse data, you can fit the constitutive relations.
