# Supplementary material for: Programming mechanics in knitted materials, stitch by stitch
Source: Nat Commun. 2024 Mar 23;15:2622. doi: 10.1038/s41467-024-46498-z (PMC10960873; doi:10.1038/s41467-024-46498-z)
Supplement: Supplementary file 3 — Source Data [file 41467_2024_46498_MOESM3_ESM.zip › SourceData/Source Data for Supplementary Information/TableS13data/TableS13.pdf]

	C^0_{xxxx} (N/mm)	C^0_{yyyy} (N/mm)	C^0_{xxyy} (N/mm)	C^0_{yyxx} (N/mm)	α_{xx}	α_{yy}	β_{xx} (N/mm)	β_{yy} (N/mm)
Lace-Weight Acrylic								
Stockinette	0.119	1.454	0.130	0.678	0.655	1.870	0.078	0.275
Garter	0.066	0.154	0.034	0.075	0.657	1.380	0.050	0.056
Rib	0.012	0.261	0.009	0.074	0.342	1.573	0.018	0.083
Seed	0.037	0.189	0.023	0.103	0.589	1.451	0.031	0.037
Lace-Weight Blue Mohair								
Stockinette	0.168	0.410	0.134	0.174	0.670	1.644	0.050	0.125
Garter	0.146	0.069	0.075	0.048	0.847	1.020	0.028	0.034
Rib	0.026	0.126	0.020	0.037	0.327	1.323	0.014	0.039
Seed	0.116	0.130	0.060	0.086	0.803	0.986	0.020	0.028
Lace-Weight Cashmere								
Stockinette	0.044	0.309	0.040	0.129	0.622	1.575	0.035	0.098
Garter	0.034	0.060	0.019	0.033	0.619	1.054	0.028	0.028
Rib	0.007	0.090	0.005	0.026	0.304	1.202	0.012	0.037
Seed	0.030	0.038	0.013	0.025	0.676	0.780	0.022	0.025
Lace-Weight Alpaca Mohair								
Stockinette	0.099	0.399	0.870	0.181	0.633	1.721	0.044	0.116
Garter	0.092	0.064	0.044	0.038	0.769	1.032	0.034	0.0311
Rib	0.021	0.152	0.018	0.051	0.389	1.476	0.012	0.051
Seed	0.104	0.076	0.048	0.052	0.817	0.869	0.018	0.026
Lace-Weight Bamboo								
Stockinette	0.015	0.432	0.016	0.186	0.605	2.095	0.012	0.103
Garter	0.023	0.068	0.015	0.037	0.638	1.542	0.018	0.022
Rib	0.005	0.100	0.004	0.030	0.323	1.787	0.008	0.028
Seed	0.019	0.043	0.010	0.025	0.669	1.019	0.014	0.021
