# Supplementary material for: Programming mechanics in knitted materials, stitch by stitch
Source: Nat Commun. 2024 Mar 23;15:2622. doi: 10.1038/s41467-024-46498-z (PMC10960873; doi:10.1038/s41467-024-46498-z)
Supplement: Supplementary file 3 — Source Data [file 41467_2024_46498_MOESM3_ESM.zip › SourceData/Source Data for Supplementary Information/TableS13data/README.rtf]

README for Table 13 Raw DataWritten by Sarah E. GonzalezLast Updated February 2 2024Included in this folder is the raw stress strain data used to make the constitutive fits described in Table 13. There is only experimental data. The data is organized by the yarn material. For example, the Lace-Weight Acrylic data is in the “acrylic” folder. The files within are organized as follows:stress in x, stress in y, strain in x, strain in y, orientation. The orientation is 0 when the fabric is pulled in the x-direction and 1 if the fabric is pulled in the y-direction.The uniaxial data can be derived from this data set by plotting stress in x versus strain in x, etc. To get the transverse data, plot stress in x versus strain in y and stress in y versus strain in x. Using all the uniaxial data and all the transverse data, you can fit the constitutive relations.
