# Supplementary material for: Programming mechanics in knitted materials, stitch by stitch
Source: Nat Commun. 2024 Mar 23;15:2622. doi: 10.1038/s41467-024-46498-z (PMC10960873; doi:10.1038/s41467-024-46498-z)
Supplement: Supplementary file 3 — Source Data [file 41467_2024_46498_MOESM3_ESM.zip › SourceData/Source Data for Supplementary Information/TableS9data/TableS9.pdf]

	C^0_{xxxx} (N/mm)	C^0_{yyyy} (N/mm)	C^0_{xxyy} (N/mm)	C^0_{yyxx} (N/mm)	α_{xx}	α_{yy}	β_{xx} (N/mm)	β_{yy} (N/mm)
Stockinette (experiment)	0.204	0.930	0.088	0.413	1.111	2.537	0.046	0.010
Stockinette (simulation)	0.200	0.753	0.040	0.341	1.391	2.971	0.005	0.013
Garter (experiment)	0.241	0.060	0.036	0.029	1.170	0.802	0.022	0.022
Garter (simulation)	0.252	0.038	0.103	0.019	1.073	1.135	0.002	0.006
Rib (experiment)	0.011	0.126	0.003	0.026	0.385	1.251	0.011	0.034
Rib (simulation)	0.024	0.142	0.011	0.028	0.446	1.411	0.004	0.014
Seed (experiment)	0.074	0.020	0.010	0.009	1.148	0.568	0.027	0.017
Seed (simulation)	0.128	0.057	0.066	0.021	0.940	0.693	0.010	0.005
