# Supplementary material for: Programming mechanics in knitted materials, stitch by stitch
Source: Nat Commun. 2024 Mar 23;15:2622. doi: 10.1038/s41467-024-46498-z (PMC10960873; doi:10.1038/s41467-024-46498-z)
Supplement: Supplementary file 3 — Source Data [file 41467_2024_46498_MOESM3_ESM.zip › SourceData/Source Data for Supplementary Information/TableS19data/TableS19.pdf]

	C^0_{xxxx} (N/mm)	C^0_{yyyy} (N/mm)	C^0_{xxyy} (N/mm)	C^0_{yyxx} (N/mm)	α_{xx}	α_{yy}	β_{xx} (N/mm)	β_{yy} (N/mm)
Stockinette	0.210	0.590	0.116	0.267	0.926	1.777	0.045	0.047
	1.225*	1.646*	0.758*	0.936*	1.806*	2.426*	0.051*	0.088*
Garter	0.149	0.076	0.035	0.032	0.928	0.732	0.043	0.039
Rib	0.046	0.452	0.028	0.100	0.483	1.547	0.039	0.059
Seed	0.108	0.120	0.225	0.044	0.889	1.006	0.047	0.028
