# Supplementary material for: Programming mechanics in knitted materials, stitch by stitch
Source: Nat Commun. 2024 Mar 23;15:2622. doi: 10.1038/s41467-024-46498-z (PMC10960873; doi:10.1038/s41467-024-46498-z)
Supplement: Supplementary file 3 — Source Data [file 41467_2024_46498_MOESM3_ESM.zip › SourceData/Source Data for Supplementary Information/TableS19data/README.rtf]

README for Table 19 Raw DataWritten by Sarah E. GonzalezLast Updated February 2 2024Included in this folder is the raw stress strain data used to make the constitutive fits described in Table 19. This folder only contains experimental data.The files within within this folder are organized as follows:stress in x, stress in y, strain in x, strain in y, orientation. The orientation is 0 when the fabric is pulled in the x-direction and 1 if the fabric is pulled in the y-direction.The uniaxial data is provided in the data for Fig S13, but it can also be derived from this data set. To get the transverse data, plot stress in x versus strain in y and stress in y versus strain in x. Using all the uniaxial data and all the transverse data, you can fit the constitutive relations.For stockinette fabric, the needle size is denoted by the diminutives ‘small’ and ‘big’ where ’small’ refers to the US size 0 needles (2.0 mm) and ‘big’ refers to the US size 2 needles (2.75 mm).
